# Prognostic factors of non-muscle invasive bladder cancer: a study based on next-generation sequencing

**DOI:** 10.1186/s12935-020-01731-9

**Published:** 2021-01-06

**Authors:** Yanxiang Shao, Xu Hu, Zhen Yang, Thongher Lia, Weixiao Yang, Kan Wu, Shangqing Ren, Sanchao Xiong, Weichao Dou, Shuyang Feng, Yaohui Wang, Yang Liu, Kang Wu, Xiang Li

**Affiliations:** 1 Department of Urology, Institute of Urology, West China Hospital, Sichuan University, 37 GuoXueXiang, Chengdu, 610041 People’s Republic of China; 2 Department of Urology, Chengdu Second People’s Hospital, Chengdu, People’s Republic of China; 3 Robot Minimally Invasive Center, Sichuan Provincial People’s Hospital, Chengdu, People’s Republic of China

**Keywords:** Bladder cancer, Bacillus Calmette–Guérin, Predictive model, Next-generation sequencing, DNA damage response and repair

## Abstract

**Objective:**

To investigate the genetic prognostic factors for the recurrence of non-muscle invasive bladder cancer.

**Materials and methods:**

The patients underwent transurethral resection of bladder tumor and received bacillus Calmette–Guérin (BCG) or epirubicin. Next-generation sequencing was performed and alterations of genes, pathways, and tumor mutation burden were recorded. Associations between these clinicopathological and genetic variants were estimated, and prognostic factor identified.

**Results:**

A total of 58 cases were included in our study, and 46 patients underwent treatment with BCG. *FGFR3* was the most frequently altered gene (48%), and more commonly detected in intermediate-risk patients. Univariate Cox analysis demonstrated that 10 genes were significantly correlated with BCG failure, while NEB, FGFR1 and SDHC were independent recurrence predictors. Besides, epigenetic-related gene pathway mutations were negatively correlated with recurrence (hazard ratio: 0.198, P = 0.023). DNA damage response and repair gene alterations were positively correlated with tumor burden, while altered TP53 was most frequent among these genes and significant correlated with high tumor burden.

**Conclusion:**

BCG instillation significantly reduced the rate of recurrence compared with epirubicin in this population. Potential biomarkers and therapeutic targets were found with the help of next-generation sequencing; correlations between DDR genes alterations and high tumor mutation burden were also demonstrated.

## Introduction

Approximately 75% of patients with bladder cancer have non-muscle invasive bladder cancer (NMIBC; Ta or T1 disease) at the time of primary diagnosis [1]. In one study, more than 70% of patients suffered recurrences in the course of treatment and surveillance, and disease progression occurred in 33% of them [2]. For these reasons, many efforts have focused on exploring prognostic factors and optimized treatments for NMIBC. Prior treatment, prior recurrence, tumor numbers, tumor size, T stage, nuclear grade and presence of carcinoma in situ (CIS) were shown to be the most important prognostic factors for NMIBC [3, 4]. The European Association of Urology (EAU) stratified these patients into three risk groups: low, intermediate, and high-risk, according to these results [5].

For intermediate- and high-risk patients, TUR-BT and subsequent intravesical drug instillation are the standard treatments. It has been confirmed that treatment with Bacillus Calmette–Guérin (BCG) following TUR-BT is superior to TUR-BT alone or combined with chemotherapy in Caucasian patients [6, 7]. However, despite the administration of maintenance therapy with BCG, 32.6% and 13.4% of patients experience recurrence and tumor progression, respectively [4].

Many attempts on bladder tumor markers have been made and markers were developed to detect tumor associated antigens, growth factors, extracellular matrix proteins [8]. Even some of them were supposed to replace urinary cytology or cystoscopy, however, no prospective data supported their impact on prognosis, especially for prediction of BCG failure [8, 9]. The revolutionary technology called next generation sequencing (NGS) enables fast and cost-effective generation of genetic sequence data with high accuracy [10]. NGS can acquire substantial information on genetic alterations, such that potential tumor markers, such as biomarkers related to failure of intravesical therapy, are more likely to be identified. Pietzak et al. used targeted NGS with cancer-associated gene panel and demonstrated that NMIBC tumors had at least one potentially actionable mutation, and DNA damage repair (DDR) gene alterations were associated with increased mutational load [11]. In an NGS study of urothelial cancer, DDR alterations were demonstrated to be independently associated with effect of immune therapy [12]. In this study, we analyzed the prognostic factors of recurrence based on a randomized controlled trial of BCG and epirubicin (EPI), examined the predictive value of prognostic models, and investigated the genetic alterations of this cohort to define the potential clinical implications of these variants.

## Patients and methods

### Patients and treatment

Patients were collected from a prospective randomized study of intravesical therapy after TUR-BT: those diagnosed as intermediate- or high-risk NMIBC were randomized to receive BCG or EPI for one-year regime by a ratio of 4:1 (phase IV clinical trial: CTR20150840 for Center for Drug Evaluation of National Medical Products Administration, China). The inclusion criteria were: age 20–75 years; TUR-BT with completely tumor resection. BT was completely resected with pathologically proven intermediate or high-risk non-muscle invasive bladder urothelium carcinoma according to the EAU guidelines for risk stratification of NMIBC (low-risk tumors were defined as primary, solitary, TaG1 (papillary urothelial neoplasm of low malignant potential and low-grade carcinomas), < 3 cm, no CIS; intermediate risk tumors were those between the category of low and high risk; characteristics of high-risk tumors were any of the following: T1, G3 tumor, CIS, multiple, recurrent and large (> 3 cm) tumors), and adjacent detrusor muscle was examined separately or together with BT [5]. Exclusion criteria were as follows: (a) Eastern Cooperative Oncology Group performance status score > 1; (b) active tuberculosis or treatment for anti-tuberculous; (c) immune deficiency or immunosuppressive therapy; (d) severe complication (e.g., serious cardiovascular and cerebrovascular disease), or presence of other types of cancer; e. previous diagnosis with muscle-invasive bladder cancer; (e) history of treatment (e.g., chemotherapy, radiotherapy or immunotherapy) during the previous 4 weeks that may influence the research results; (f) serious intraoperative and postoperative complications (e.g., bladder perforation, serious postoperative hematuria, bladder irritation, etc.); and (g) contraindications to treatment or inability to participate in our trial due to pregnancy, severe disability, or serious psychological problems.

### Follow-up and outcomes

For intermediate-risk patients, cystoscopy was performed 3 and 6 months after surgery, then biannually for 5 years. For high-risk patients, cystoscopy was performed trimonthly until 2 years after surgery, then biannually for 5 years. The initiation time was the day TUR-BT was performed. The primary end point was recurrence during follow-up, while the secondary end point was progression to muscle-invasive bladder cancer or therapy-related discontinuation of treatment. Recurrence-free survival (RFS) and progression-free survival (PFS) times were recorded. BCG failure and intolerance were recorded according to the definitions of the EAU [5].

### Data collection

Clinicopathological variables included gender, age, tumor size, site, number, tumor histopathological type, T stage (8th American Joint Committee on Cancer TNM classification system) [13], tumor grade (2004 World Health Organization grading system), and CIS status. All pathological reports were provided by the Pathology Department of West China Hospital, Sichuan University.

Paraffin-embedded tissues obtained from the BCG treatment group were collected for NGS. Patients provided written informed consent. Sequencing was performed on a Nextseq 500 sequencer (Illumina, Inc., San Diego, CA, USA) for 520 cancer-related genes, including the whole exons of 312 genes, as well as critical exons, introns, and promoter regions of the remaining 208 genes. Resultant sequences were analyzed for gene alternations. Nonsynonymous mutations, deletions, nonsense mutations, transcoding mutations, and splicing mutations were considered significant. In addition, the tumor mutation burden (TMB) was also recorded. Genomic pathways were analyzed in qualitative and quantitative dimensions (i.e., whether mutations are present and the number of mutations genes in each pathway).

### Statistical methods

One-way analysis of variance and the Chi-squared test were used for comparisons between groups. The Chi-squared test was used to analyze categorical variants. The Pearson Chi-squared test was used when n ≥ 40 and T ≥ 5; the continuously correcting Chi-squared test was used when n ≥ 40 and 1 ≤ T<5; if n < 40 or T < 0, Fisher’s exact test was used. Kruskal–Wallis and Wilcoxon tests were used to analyze continuous variants. RFS and PFS were estimated using the Kaplan–Meier method. Differences in survival between two or more subgroups were evaluated using log-rank tests. Univariate and multivariate Cox regression analyses were performed to determine the clinicopathological parameters associated with the recurrence of patients with NMIBC. Patients were stratified using the European Organisation for Research and Treatment of Cancer (EORTC) risk tables [3] and the Spanish Urological Club for Oncological Treatment (CUETO) scoring model [4].

Statistical analyses were performed using the SPSS Statistics version 25 (IBM, Armonk, NY, USA). P-value < 0.05 denoted statistical significance.

## Results

### Patient baseline data and prognosis

The clinicopathological data from 58 patients are shown in Table 1. The mean age was 61.91 years (standard deviation, SD: 7.67 years), and the median follow-up time was 32.86 months (interquartile range, IQR: 21.00–44.49 months). There were no significant differences between BCG and EPI subgroups in terms of baseline clinicopathological data except for T stage. For the BCG subgroup, recurrence occurred in nine patients, and six of these recurred during course of intravesical BCG therapy. Two-year RFS rate for the BCG group was 82.2%; while for EPI subgroup, seven patients experienced recurrences, five of whom did so during the EPI treatment course. The two-year RFS rate for EPI was 58.3%. Univariate analysis revealed significantly better RFS in the BCG-treated subgroup (P = 0.020) and in those with incipient tumor (P = 0.001 for all 58 patients and P = 0.014 for 46 BCG-treated patients) (Additional file 1: Table S1).

**Table 1:** Clinicopathological data of patients with NMIBC

Clinicopathological variants	BCG group (n = 46)	EPI group (n = 12)	Total (n = 58)	P-value
Gender				0.432
Male	35	11	46	
Female	11	1	12	
Preoperative age (years)				0.336
≤ 60	18	7	25	
60–70	19	4	23	
> 70	9	1	10	
Smoking history				0.331
No	25	4	29	
Yes	21	8	29	
Prior recurrence rate				0.233
Primary	27	5	32	
Recurrent, ≤ 1 rec/year	11	2	13	
Recurrent, > 1 rec/year	8	5	13	
T category				0.012
Ta	29	12	41	
T1	17	0	17	
Grade				0.331
Low grade	21	8	29	
High grade	25	4	29	
Tumor size (cm)				0.759
< 3	26	8	34	
≥ 3	20	4	24	
No. tumors				0.891
≤ 3	34	8	42	
> 3	12	4	16	
Risk stage				0.291
Intermediate risk	19	5	26	
High risk	27	5	32	
EORTC risk tables				0.956
1–4	17	3	20	
5–9	24	9	33	
10–17	5	0	5	
CUETO scoring model				0. 866
0–4	13	3	16	
5–6	16	5	21	
7–9	15	4	19	
10–16	2	0	2	

NMIBC: non-muscle invasive bladder cancer; n: number of patients; No: number; Recurrent, ≤ 1 rec/year: prior recurrence rate of less than one per year; Recurrent, > 1 rec/year: prior recurrence rate of more than one per year; EORTC: European Organization for Research and Treatment of Cancer; CUETO: Spanish Urological Club for Oncological Treatment

### Genomic landscape of patients with NMIBC and relationship with prognosis

NGS was performed on the total 58 patients, the most frequently altered genes were *FGFR3* (48%), *KDM6A* (47%), *KMT2D* (43%), *KMT2C* (34%), and *STAG2* (31%). Oncoprints for these genes are presented in Additional file 2, Figures S1–S7). Significant mutations of *FGFR3* were observed in 73.1% of intermediate-risk patients (19/26) and 15.6% of high-risk patients (5/32). For *STAG2*, these numbers were 46.2% (12/26) and 18.8% (6/32), respectively. Mutations of *FGFR3*, *STAG2*, and *PRKDC* were significantly associated with tumor risk stage (P < 0.05, Additional file 1: Table S2). Mutations of 17 genes (such as *KDM6A* and *ARID1A*) significant correlated with higher EORTC scores, while mutations of other 17 genes (such as TP53) significantly correlated with higher CUETO scores (Additional file 1: Table S3).

Cox univariate analysis was performed to identify correlations of genomic mutations with recurrence. In all 58 cases, 18 genes were significantly correlated with recurrence (Table 2 and Additional file 1: Table S4); all these mutations were positively associated with poor outcome. However, among these genes, only mutations of *NEB*, *MLH1*, *GATA3*, *FGFR1* and *RAF1* occurred in > 5% of patients. Similar results were observed in the BCG subgroup (46 cases), with ten genes significantly associated with recurrence (P < 0.05, Table 2 and Additional file 1: Table S5). The multivariate Cox analysis included all genes significantly associated with recurrence, the results demonstrated that *NEB*, *MLH1*, *FGF12,* and *FGFR1* independently predicted poor outcome in all 58 patients (P = 0.001, 0.001, 0.001, and 0.007, respectively), while *NEB*, *FGFR1* and *SDHC* were independent prognostic predictor for recurrence in patients treated with BCG (P = 0.001, 0.004 and 0.017 respectively) (Additional file 1: Table S6).

**Table 2:** Univariate analysis of genetic mutations and recurrence survival rate

Gene	Alterations (%)	Total patients (n = 58)		BCG subgroup (n = 46)	
		P-value	Hazard ratio (95% CI)	P-value	Hazard ratio (95% CI)
*NEB*	5.2	0.001	17.307 (3.305–90.639)	0.001	21.213 (3.466–129.823)
*MLH1*	5.2	0.011	18.580 (1.933–178.630)	NA	NA
*CDKN1B*	3.4	0.011	18.580 (1.933–178.630)	NA	NA
*FGF12*	1.7	0.011	18.580 (1.933–178.630)	0.012	21.995 (1.994–242.587)
*NKX2_1*	1.7	0.011	18.580 (1.933–178.630)	NA	NA
*RET*	1.7	0.011	18.580 (1.933–178.630)	NA	NA
*SDHB*	1.7	0.011	18.580 (1.933–178.630)	NA	NA
*TAF1*	1.7	0.011	18.580 (1.933–178.630)	0.012	14.493 (1.507–139.347)
*EPHB1*	3.4	0.019	13.794 (1.542–123.422)	NA	NA
*PIK3C3*	1.7	0.019	13.794 (1.542–123.422)	NA	NA
*ZNF703*	1.7	0.019	13.794 (1.542–123.422)	NA	NA
*PALB2*	3.4	0.028	5.421 (1.195–24.597)	0.056	8.100 (0.946–69.384)
*GATA3*	5.2	0.024	6.140 (1.263–29.846)	0.178	4.394 (0.510–37.818)
*FGFR1*	5.2	0.029	10.924 (1.276–93.521)	0.021	14.493 (1.507–139.347)
*MAX*	1.7	0.029	10.924 (1.276–93.521)	0.021	14.493 (1.507–139.347)
*SDHC*	12.1	0.074	3.917 (0.877–17.507)	0.003	11.892 (2.272–62.246)
*MTOR*	5.2	0.052	4.442 (0.989–19.956)	0.024	6.301 (1.278–31.072)
*RAF1*	5.2	0.042	8.986 (1.082–74.659)	0.034	10.686 (1.194–95.634)
*RECQL4*	1.7	0.042	8.986 (1.082–74.659)	0.034	10.686 (1.194–95.634)
*SDHAF2*	1.7	0.042	8.986 (1.082–74.659)	0.034	10.686 (1.194–95.634)

Mutated genes significantly correlated with prognosis are shown. BCG: Bacillus Calmette–Guérin; CI: confidence interval; n: number; NA: not available

### Genomic pathways and recurrence

**Fig. 1:**
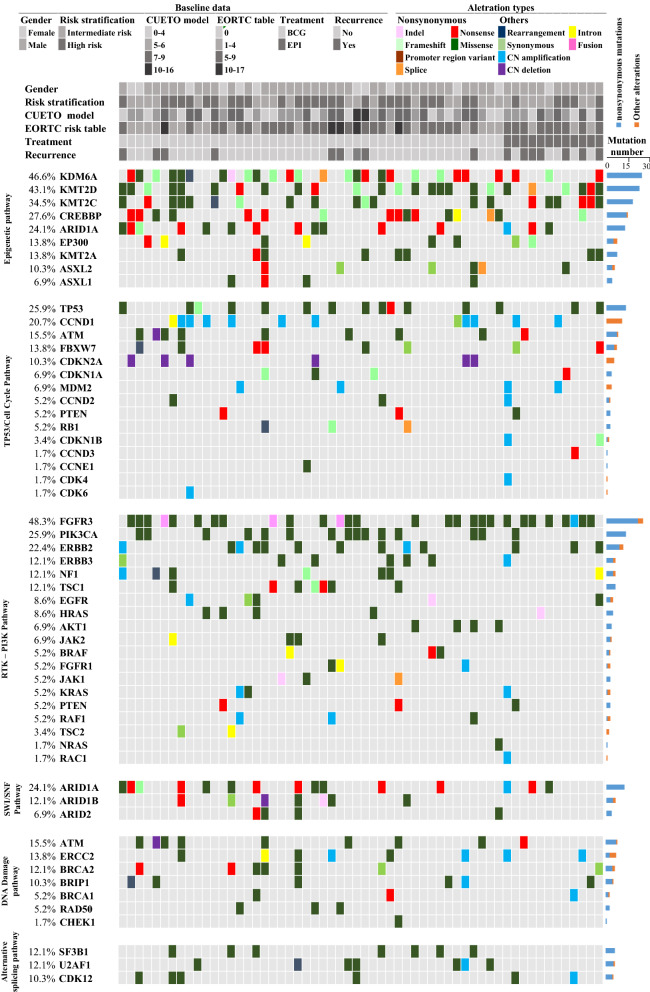
Oncoprint of genetic pathways. BCG: Bacillus Calmette–Guérin treated group; EPI: epirubicin treated group. CUETO model: Spanish Urological Club for Oncological Treatment scoring model; EORTC: European Organization for Research and Treatment of Cancer; CN: copy number; RTK: receptor tyrosine kinases; PI3K: phosphatidylinositol 3-kinase; SWI/SNF: Switch/sucrose nonfermentable

Canonical genomic pathways were analyzed to define whether mutations were associated with prognosis (Fig. 1). Mutations of the receptor tyrosine kinase/RAS/phosphatidylinositol 3-kinase (RTK/RAS/PI3K) pathway were found in 87.9% of patients, and were associated with higher risk stage (P = 0.013, Additional file 1: Table S7). Epigenetic-related gene mutations were also frequently detected in patients with NMIBC (49/58, 84.4%), with markedly lower rates of mutations in the TP53/cell cycle pathway, the switch/sucrose nonfermentable pathway, the DNA damage pathway, and the alternative splicing pathway (50.0%, 31.0%, 32.8%, and 34.5%, respectively). Correlations between the number of pathway mutations and prediction models were also analyzed: larger numbers of mutated genes in the epigenetic pathway, histone modification pathway, and the switch/sucrose nonfermentable pathway significantly correlated with a poorer EORTC score, whereas there was no significant correlation with the CUETO score (Additional file 1: Table S7).

Correlations of pathway mutations with recurrence were analyzed using Cox regression in qualitative and quantitative dimensions (Table 3). In qualitative analysis, mutations in epigenetic-related genes, the RTK-PI3K pathway, the SWI/SNF pathway and the alternative splicing pathway were protective factors (hazard ratio [HR] < 1), while TP53/cell cycle pathway mutations were risk factor, in all 58 patients and in the BCG subgroup. Mutations in epigenetic-related genes were significantly associated with recurrence in qualitative analysis (P = 0.020 and 0.023 for total patients and for the BCG subgroup, respectively). Nevertheless, when quantitative analysis was performed, we found that no pathway mutations significantly correlated with recurrence (P > 0.05, Table 3).

**Table 3:** Univariate analysis of mutated genetic pathways, DDR genes, and recurrence survival rate

Genetic pathway	Total patients (n = 58)		BCG subgroup (n = 46)	
	P-value	Hazard ratio (95% CI)	P-value	Hazard ratio (95% CI)
Qualitative				
Epigenetic-related genes	0.020	0.278 (0.095–0.814)	0.023	0.198 (0.049–0.797)
TP53/cell cycle pathway	0.668	0.805 (0.299–2.170)	0.725	0.790 (0.212–2.942)
RTK-PI3K pathway	0.830	0.849 (0.192–3.765)	0.351	0.473 (0.098–2.280)
SWI/SNF pathway	0.182	0.424 (0.120–1.497)	0.397	0.507 (0.105–2.439)
DNA damage pathway	0.554	0.710 (0.229–2.204)	0.965	0.969 (0.242–3.878)
Alternative splicing pathway	0.059	0.240 (0.054–1.056)	0.289	0.427 (0.089–2.056)
DDR genes	0.298	2.931 (0.387–22.211)	0.632	1.662 (0.208–13.306)
Quantitative				
Epigenetic-related genes	0.960	0.993 (0.756–1.303)	0.877	1.030 (0.710–1.494)
TP53/cell cycle pathway	0.942	1.016 (0.665–1.553)	0.958	0.985 (0.566–1.716)
RTK-PI3K pathway	0.096	0.686 (0.440–1.069)	0.308	0.756 (0.441–1.295)
SWI/SNF pathway	0.170	0.567 (0.252–1.275)	0.379	0.671 (0.276–1.632)
DNA damage pathway	0.319	0.700 (0.348–1.411)	0.582	0.810 (0.382–1.716)
Alternative splicing pathway	0.101	0.400 (0.134–1.195)	0.410	0.629 (0.208–1.897)
DDR genes	0.603	0.951 (0.788–1.148)	0.808	0.974 (0.789–1.203)

DDR: DNA damage response and repair; BCG: Bacillus Calmette–Guérin; CI: confidence interval; RTK: receptor tyrosine kinases; PI3K: phosphatidylinositol 3-kinase; SWI/SNF: Switch/sucrose nonfermentable; n: number

### DDR genes and TMB

DDR genes have been associated with increased mutation load and with the effect of immunotherapy against urothelial carcinoma [12]. We also analyzed the correlation between DDR, TMB, and prognosis (the list of DDR genes is provided in Additional file 1: Table S8 and the alteration status is shown in Additional file 2: Figure S8). The percentages of DDR mutations in 58 cases with NMIBC and BCG subgroups were 86.2% and 84.8%, respectively (numbers of mutations were 165 and 141, respectively). *TP53* was the most frequently mutated gene among the DDR genes (19/165 in the total cases and 17/141 in the BCG subgroups). Of note, missense variant was the most common alteration in *TP53* (16/19 and 14/17 in the total and BCG group, respectively) (Additional file 3: Figure S8). In our previous analysis, *TP53* mutation correlated with poorer CUETO score; however, there were no correlations with tumor risk group stratification, EORTC score, or RFS (Additional file 1: Tables S2–5). When considering mutations in all DDR genes, there were no correlations demonstrated with the predicted models or RFS (P > 0.05) (Table 3 and Additional file 1: Table S7).

**Fig. 2:**
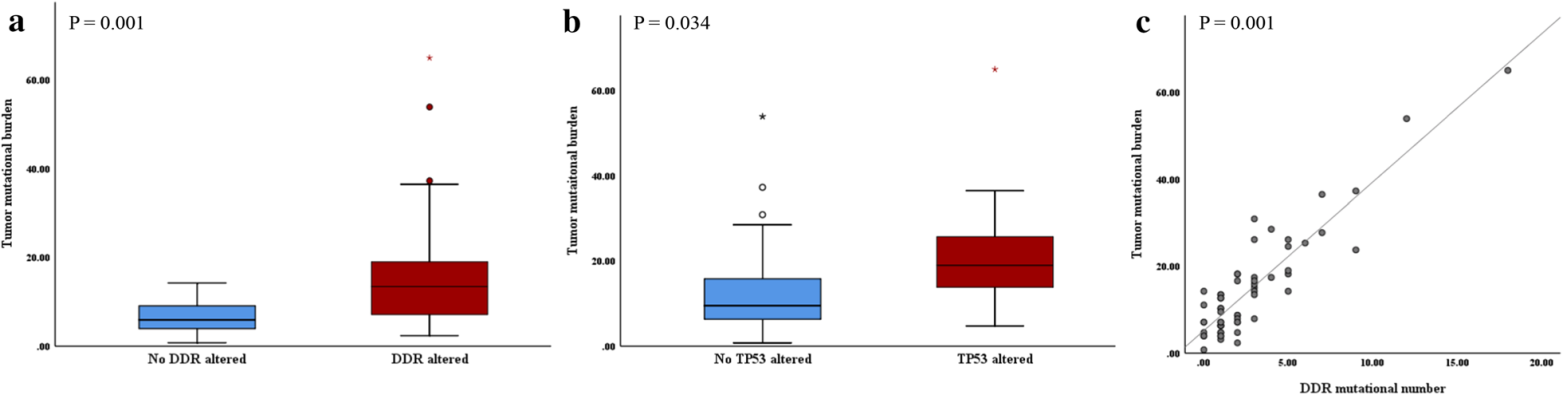
Correlation analysis between DNA damage response and repair genes and TMB. **a** Correlation of the TMB and DDR alteration status. **b** Correlation of the TMB and DDR mutational numbers. DDR: DNA damage response and repair. **c** Correlation of the TMB and *TP53* alteration status. TMB: tumor mutational burden

In the analysis of TMB, significant correlations were found with the EORTC model (P-values for correlation with risk group stratification, CUETO, and EORTC scores were 0.485, 0.706, and 0.037, respectively). There was no significant correlation between TMB and RFS (HR: 0.972, P = 0.316 for 58 NMIBC cases and HR: 0.976, P = 0.497 for 46 BCG subgroup cases). However, significant correlations were demonstrated between alterations in DDR and TMB, and between DDR mutated numbers and TMB (Figs. 2a, b). In addition, TP53 mutations were also significantly associated with TMB (Fig. 2c).

## Discussion

Recurrence of NMIBC is affected by numerous factors, including tumor size, number of tumors, prior recurrence rate, T stage, grade, and presence of CIS [3]. Since first reported, the efficacy of BCG irrigation has been widely demonstrated and became the standard therapy for intermediate- and high-risk NMIBC [14]. However, recurrence continues to occur in many patients treated with BCG; 32.6% as reported by Jesus et al. [4]. Even after a full course of maintenance therapy with BCG, 15.7% and 26.3% of patients continue to experience early and late recurrence, respectively [15].

Pretreatment biomarkers can help to predict the therapeutic effect of BCG against NMIBC [2]. In fact, these biomarkers can be used as predictors and carry therapeutic value. For example, the protein encoded by the *MTOR* gene belongs to the family of phosphatidylinositol kinases, which mediate DNA damage and nutrient deficiency [16]. Hansel et al. reported that abnormal activation of phosphorylated *MTOR* and its downstream target was associated with increased pathological stage and poorer survival [17]. In our study, we also found a correlation between *MTOR* mutations and tumor recurrence. Therefore, we believe that *MTOR* can serve as a potential biomarker for recurrence of NMIBC. In addition, it was reported that inhibition of *MTOR* expression induced apoptosis of bladder cancer cells and inhibited cell cycle progression, cell growth, angiogenesis, and endothelial cell proliferation, indicating its therapeutic value [18]. In fact, MTOR protein is a key downstream protein kinase in the PI3K/AKT signaling pathway, with mutations occurring in approximately 40% of bladder cancer cases [19]. However, there was no correlation found between mutations of the RTK-PI3K pathway and recurrence in our study, and the underlying mechanism warrants further research.

Epigenetic regulations, which include DNA methylation, histone modification and chromatin remodeling, control gene expression and tumor biology [20]. Alterations of histone modification occurred in nearly 89% of invasive bladder cancer cases [21], while DNA hypermethylation correlated with grade and stage [22, 23]. In vitro, tumor suppressor genes can be restored after treatment with DNA methyltransferases and histone deacetylase [24, 25], suggesting the therapeutic significance of the epigenetic pathway in bladder cancer. Moreover, chromatin remodeling genes, such as *ARID1A* and *KDM6A* were frequently mutated in bladder cancer [21, 26]. Pietzak et al., found that mutated *ARID1A* correlated with recurrence and could be used as a biomarker [27]. However, in our study, a negative correlation was found between mutations of epigenetic-related genes and recurrence, and there was no prognostic significance demonstrated for *ARID1A*. In fact, epigenetic dysregulations occur in different stages of various tumors; this can be either a cause or an effect of tumor recurrence and progression. Further studies of the underlying mechanism are warranted to understand the significance of epigenetic regulation in NMIBC.

In our study, mutated *FGFR1* and *NEB* were shown to be independent predictors of recurrence, despite their low frequency of alterations observed in the present cohort (5.2%). Rehab et al. found that overexpression of *FGFR1* was common in malignant bladder cancer, and the sensitivity and specificity of combined *FGFR1* and *CK20* for the differentiation between MIBC and NMIBC can be as high as 97.5% and 92.5%, respectively [28]. These results suggest that *FGFR1* can serve as a biomarker of tumor invasiveness. Although we believe this is also the case for *NEB*, further investigations are warranted. However, except for the aforementioned independent predictors, we did not consider all the statistically significant gene mutations with low mutation rates as clinically significant.

The clinical importance of some genes could not be ignored even in the absence of prognostic significance. The telomerase reverse transcriptase gene (*TERT*) is involved in DNA protection and cellular ageing processes [29], and mutations of this gene occur in > 70% of bladder cancer cases [27, 30]. Although there was no correlation found between *TERT* mutation and tumor stage, grade, and prognosis in our and previous studies, this gene was also considered a potential biomarker for screening and surveillance owing to its high mutation rate in patients with bladder cancer [30, 31]. *FGFR3* is also clinically significant, whose mutations have been identified as a key to tumor initiation [32]. *FGFR3* can serve as a potential biomarker, based on the negative correlation of mutated *FGFR3* and tumor malignant degree. *FGFR3* mutations were more commonly found in NMIBC than MIBC in a previous study [11]. In our study, these mutations were also more common in the intermediate-risk than the high-risk NMIBC group. *FGFR3* is also a potential treatment target as it can activate many downstream kinase pathways, including the RTK/RAS pathway [32]. The antitumor activity of BGJ398, a selective inhibitor of *FGFR*, has been demonstrated in patients with FGFR3 mutations [33]. There is an ongoing clinical trial of BGJ398 in patients with NMIBC, and results are expected in the future.

DDR-related genes were frequently altered in high-grade NMIBC [27], and these alterations were associated with increased mutation burden and better efficacy of chemotherapy and immunotherapy [12, 34]. Min et al. studied DDR alterations in 60 patients with advanced urothelial cancer treated with adjuvant PD-1/PD-L1 blockade therapy, they found that a higher response rate to immune therapy in the DDR-altered group (67.9% vs. 18.8%), as well as longer PFS and overall survival [12]. Therefore, we believe that DDR alterations may influence the efficacy of BCG instillation, also based on the activation of the autoimmune system. Nevertheless, we found that DDR alterations were only significantly correlated with TMB and there was no correlation with risk stage or prognosis.

*TP53* was the most frequently mutated DDR gene in our study. DNA damage can trigger the activation of p53, which maintains genetic stability and controls the transcription of DNA repair-related genes [35]. A recent meta-analysis suggested that p53 overexpression in NMIBC might be associated with poor RFS in an Asian population [36]. However, there was no prognostic significance of *TP53* mutation or the mutated TP53/cell cycle pathway observed in our study. Interestingly, mutated *TP53* gene was associated with larger TMB, while these two variants correlated with higher CUETO and EORTC scores. This evidence suggests that prognostic models may serve as bridges for understanding the correlation between DDR alterations and NMIBC recurrence.

There were some limitations in our study. Although it was based on a randomized controlled trial with limited heterogeneity among the subjects, the sample size was relatively small for the analysis of the association between prognosis and various clinicopathological and genomic variants. Furthermore, the follow-up period was limited, with a small number of patients reaching the follow-up endpoint; therefore, PFS and overall survival could not be calculated.

## Conclusion

Maintenance treatment with BCG provided better control of recurrence in Chinese patients with NMIBC after TUR-BT versus intravesical therapy with EPI. NGS of these patients showed that *FGFR1* and *NEB* are biomarkers of recurrence, while mutations in the *MTOR* gene and epigenetic pathway are potential treatment targets for patients who fail treatment with BCG. DDR alterations were positively correlated with higher TMB, *TP53* may be the key to understanding the relationship between DDR and recurrence after treatment with BCG (Additional file 4).

## Supplementary information

**Additional file 1:** Tables S1 to S8.

**Additional file 2:** Figures S1 to S7.

**Additional file 3:** Figure S8.

**Additional file 4:** Original dataset.

## Data Availability

The dataset supporting the conclusions of this article is included within the article and its supplementary materials.
