# Supplementary material for: Prognostic factors of non-muscle invasive bladder cancer: a study based on next-generation sequencing
Source: Cancer Cell Int. 2021 Jan 6;21:23. doi: 10.1186/s12935-020-01731-9 (PMC7789352; doi:10.1186/s12935-020-01731-9)
Supplement: Supplementary file 1 — Additional file 1: Tables S1 to S8. [file 12935_2020_1731_MOESM1_ESM.docx]

**Prognostic factors of non-muscle invasive bladder cancer: A study based on next-generation sequencing**

**Catalogue**

**Table S1………………………………………………………………………….. 3**

**Table S2………………………………………………………………………….. 4**

**Table S3………………………………………………………………………….. 12**

**Table S4………………………………………………………………………….. 20**

**Table S5………………………………………………………………………….. 28**

**Table S6………………………………………………………………………….. 36**

**Table S7………………………………………………………………………….. 37**

**Table S8………………………………………………………………………….. 38**

**Supplementary figure Legends ……………………………………………….. 39**

**Table S1.** Univariate analysis of the NMIBC cohort and BCG subgroup

| Clinical pathological data | Total patients |  | BCG subgroup |  |
| --- | --- | --- | --- | --- |
|  | P value | Hazard ratio (95% CI) | P value | Hazard ratio (95% CI) |
| Females^a^ | 0.407 | 1.872 (0.425 - 8.239) | 0.911 | 1.094 (0.227–5.267) |
| 60–70 years^b^ | 0.173 | 0.466 (0.155 - 1.397) | 0.542 | 0.627 (0.140–2.806) |
| >70 years^c^ | 0.316 | 0.456 (0.098 - 2.12) | 0.961 | 0.958 (0.175–5.233) |
| Smoking history^d^ | 0.308 | 1.693 (0.615 - 4.661) | 0.536 | 1.515 (0.407–5.642) |
| Recurrent^e^ | 0.001 | 12.547 (2.82 - 55.829) | 0.014 | 13.747 (1.717–110.042) |
| Recurrent, ≤1 rec/yr^f^ | 0.041 | 5.925 (1.074 - 32.703) | 0.079 | 7.611 (0.791–73.185) |
| Recurrent, >1 rec/yr^g^ | 0.001 | 21.364 (4.652 - 98.119) | 0.006 | 27.133 (3.149–233.777) |
| T1^h^ | 0.670 | 1.261 (0.434 - 3.657) | 0.151 | 2.623 (0.703–9.789) |
| High grade^i^ | 0.303 | 0.587 (0.213 - 1.619) | 0.899 | 1.089 (0.292–4.057) |
| Tumor size ≥3 cm^j^ | 0.018 | 0.166 (0.038 - 0.732) | 0.059 | 0.135 (0.017–1.081) |
| No. tumors >3^k^ | 0.129 | 2.151 (0.799 - 5.792) | 0.173 | 2.498 (0.670–9.312) |
| High-risk tumor stage^l^ | 0.831 | 1.114 (0.414 - 2.994) | 0.229 | 2.622 (0.545–12.625) |
| EPI group^n^ | 0.020 | 3.234 (1.203 - 8.692) | - | - |

^a^: compared with males; ^b^ and ^c^: compared with age ≤60 years; ^d^: compared with non-smokers; ^e^, ^f^, and ^g^: compared with incipient patients; ^h^: compared with Ta; ^i^: compared with low grade; ^j^: compared with tumor size <3 cm; ^k^: compared with number of tumors ≤3; ^l^: compared with intermediate-risk patients; ^m^: compared with the BCG 19 group; ^n^: compared with the BCG cohort.

NMIBC: non-muscle invasive bladder cancer; BCG: Bacillus Calmette–Guérin; CI: confidence interval.; Recurrent, ≤1 rec/yr: prior recurrence rate of less than one per year; Recurrent, >1 rec/yr: prior recurrence rate of more than one per year.

**Table S2.** Correlation between single genetic alterations and NMIBC risk stage

| Gene | P value | Odds risk | Lower limit of 95% CI | Upper limit of 95% CI |
| --- | --- | --- | --- | --- |
| *FGFR3* | 9.96E-06 | 0.068226 | 0.018799383 | 0.247604062 |
| *STAG2* | 0.024866 | 0.269231 | 0.083077175 | 0.872504475 |
| *PRKDC* | 0.028077 | 0.5 | 0.381004047 | 0.656161009 |
| *TP53* | 0.051886 | 4.6 | 1.134467286 | 18.65192612 |
| *ARID1B* | 0.058304 | 0.509434 | 0.391157816 | 0.663473799 |
| *ERBB3* | 0.058304 | 0.509434 | 0.391157816 | 0.663473799 |
| *ATRX* | 0.084262 | 0.418182 | 0.306184197 | 0.571146502 |
| *BCORL1* | 0.084262 | 0.418182 | 0.306184197 | 0.571146502 |
| *POLE* | 0.084262 | 0.418182 | 0.306184197 | 0.571146502 |
| *RAD54L* | 0.084262 | 0.418182 | 0.306184197 | 0.571146502 |
| *U2AF1* | 0.116529 | 0.135484 | 0.014753981 | 1.244130625 |
| *CSF1R* | 0.119994 | 0.518519 | 0.400999631 | 0.670478059 |
| *ERBB2* | 0.16578 | 4 | 0.768541179 | 20.81866325 |
| *CREBBP* | 0.169973 | 0.435897 | 0.131246665 | 1.447705928 |
| *TSC1* | 0.184336 | 5.769231 | 0.647592791 | 51.39653206 |
| *DDR2* | 0.196612 | 0.428571 | 0.316723265 | 0.579917834 |
| *PTPN11* | 0.196612 | 0.428571 | 0.316723265 | 0.579917834 |
| *CTCF* | 0.196612 | 0.428571 | 0.316723265 | 0.579917834 |
| *PTPRT* | 0.196612 | 0.428571 | 0.316723265 | 0.579917834 |
| *WT1* | 0.196612 | 0.428571 | 0.316723265 | 0.579917834 |
| *BCOR* | 0.196612 | 0.428571 | 0.316723265 | 0.579917834 |
| *PALB2* | 0.196612 | 0.428571 | 0.316723265 | 0.579917834 |
| *PIK3C2G* | 0.196612 | 0.428571 | 0.316723265 | 0.579917834 |
| *EP300* | 0.236412 | 0.177419 | 0.018543512 | 1.697500831 |
| *BRIP1* | 0.236412 | 0.177419 | 0.018543512 | 1.697500831 |
| *HRAS* | 0.236412 | 0.177419 | 0.018543512 | 1.697500831 |
| *ERCC2* | 0.245009 | 0.527273 | 0.410542417 | 0.677193189 |
| *SDHC* | 0.245009 | 0.527273 | 0.410542417 | 0.677193189 |
| *ERBB4* | 0.245009 | 0.527273 | 0.410542417 | 0.677193189 |
| *FLT4* | 0.245009 | 0.527273 | 0.410542417 | 0.677193189 |
| *PTPRD* | 0.245009 | 0.527273 | 0.410542417 | 0.677193189 |
| *ABL2* | 0.245009 | 0.527273 | 0.410542417 | 0.677193189 |
| *IDH1* | 0.245009 | 0.527273 | 0.410542417 | 0.677193189 |
| *JAK1* | 0.245009 | 0.527273 | 0.410542417 | 0.677193189 |
| *MSH3* | 0.245009 | 0.527273 | 0.410542417 | 0.677193189 |
| *RAD50* | 0.245009 | 0.527273 | 0.410542417 | 0.677193189 |
| *SPEN* | 0.245009 | 0.527273 | 0.410542417 | 0.677193189 |
| *FANCA* | 0.263148 | 3.36 | 0.63358566 | 17.8185851 |
| *TERT* | 0.335148 | 2.555556 | 0.602519662 | 10.83925491 |
| *LRP1B* | 0.405582 | 2.769231 | 0.509065667 | 15.06414507 |
| *ARID1A* | 0.431156 | 1.643478 | 0.474168571 | 5.696330297 |
| *FGF3* | 0.448276 | 0.438596 | 0.326971144 | 0.588329844 |
| *SH2B3* | 0.448276 | 0.438596 | 0.326971144 | 0.588329844 |
| *EMSY* | 0.448276 | 0.438596 | 0.326971144 | 0.588329844 |
| *FANCD2* | 0.448276 | 0.438596 | 0.326971144 | 0.588329844 |
| *KDM5A* | 0.448276 | 0.438596 | 0.326971144 | 0.588329844 |
| *MRE11* | 0.448276 | 0.438596 | 0.326971144 | 0.588329844 |
| *RUNX1T1* | 0.448276 | 0.438596 | 0.326971144 | 0.588329844 |
| *TNFAIP3* | 0.448276 | 0.438596 | 0.326971144 | 0.588329844 |
| *ALK* | 0.448276 | 0.438596 | 0.326971144 | 0.588329844 |
| *ANKRD11* | 0.448276 | 0.438596 | 0.326971144 | 0.588329844 |
| *CARD11* | 0.448276 | 0.438596 | 0.326971144 | 0.588329844 |
| *CRLF2* | 0.448276 | 0.438596 | 0.326971144 | 0.588329844 |
| *GRIN2A* | 0.448276 | 0.438596 | 0.326971144 | 0.588329844 |
| *MST1* | 0.448276 | 0.438596 | 0.326971144 | 0.588329844 |
| *NOTCH2* | 0.448276 | 0.438596 | 0.326971144 | 0.588329844 |
| *NTRK1* | 0.448276 | 0.438596 | 0.326971144 | 0.588329844 |
| *PDGFRB* | 0.448276 | 0.438596 | 0.326971144 | 0.588329844 |
| *PIK3R2* | 0.448276 | 0.438596 | 0.326971144 | 0.588329844 |
| *PPP2R1A* | 0.448276 | 0.438596 | 0.326971144 | 0.588329844 |
| *PRKAR1A* | 0.448276 | 0.438596 | 0.326971144 | 0.588329844 |
| *RAD51* | 0.448276 | 0.438596 | 0.326971144 | 0.588329844 |
| *TIPARP* | 0.448276 | 0.438596 | 0.326971144 | 0.588329844 |
| *ZFHX3* | 0.448276 | 0.438596 | 0.326971144 | 0.588329844 |
| *ARID5B* | 0.448276 | 0.438596 | 0.326971144 | 0.588329844 |
| *B2M* | 0.448276 | 0.438596 | 0.326971144 | 0.588329844 |
| *BCL10* | 0.448276 | 0.438596 | 0.326971144 | 0.588329844 |
| *CEBPA* | 0.448276 | 0.438596 | 0.326971144 | 0.588329844 |
| *CSF3R* | 0.448276 | 0.438596 | 0.326971144 | 0.588329844 |
| *CXCR4* | 0.448276 | 0.438596 | 0.326971144 | 0.588329844 |
| *DNAJB1* | 0.448276 | 0.438596 | 0.326971144 | 0.588329844 |
| *ERCC4* | 0.448276 | 0.438596 | 0.326971144 | 0.588329844 |
| *FANCE* | 0.448276 | 0.438596 | 0.326971144 | 0.588329844 |
| *FANCF* | 0.448276 | 0.438596 | 0.326971144 | 0.588329844 |
| *FGFR4* | 0.448276 | 0.438596 | 0.326971144 | 0.588329844 |
| *GNAQ* | 0.448276 | 0.438596 | 0.326971144 | 0.588329844 |
| *H3F3C* | 0.448276 | 0.438596 | 0.326971144 | 0.588329844 |
| *HGF* | 0.448276 | 0.438596 | 0.326971144 | 0.588329844 |
| *HIST1H3J* | 0.448276 | 0.438596 | 0.326971144 | 0.588329844 |
| *IGF2* | 0.448276 | 0.438596 | 0.326971144 | 0.588329844 |
| *KAT6A* | 0.448276 | 0.438596 | 0.326971144 | 0.588329844 |
| *MAGI2* | 0.448276 | 0.438596 | 0.326971144 | 0.588329844 |
| *MAP3K13* | 0.448276 | 0.438596 | 0.326971144 | 0.588329844 |
| *MSH6* | 0.448276 | 0.438596 | 0.326971144 | 0.588329844 |
| *NRAS* | 0.448276 | 0.438596 | 0.326971144 | 0.588329844 |
| *NTRK3* | 0.448276 | 0.438596 | 0.326971144 | 0.588329844 |
| *PAX5* | 0.448276 | 0.438596 | 0.326971144 | 0.588329844 |
| *POLD1* | 0.448276 | 0.438596 | 0.326971144 | 0.588329844 |
| *RHOA* | 0.448276 | 0.438596 | 0.326971144 | 0.588329844 |
| *RIT1* | 0.448276 | 0.438596 | 0.326971144 | 0.588329844 |
| *SMARCD1* | 0.448276 | 0.438596 | 0.326971144 | 0.588329844 |
| *TNFSF11* | 0.448276 | 0.438596 | 0.326971144 | 0.588329844 |
| *WRN* | 0.448276 | 0.438596 | 0.326971144 | 0.588329844 |
| *XIAP* | 0.448276 | 0.438596 | 0.326971144 | 0.588329844 |
| *SETD2* | 0.461389 | 0.247312 | 0.024145298 | 2.533128435 |
| *AKT1* | 0.461389 | 0.247312 | 0.024145298 | 2.533128435 |
| *ARID2* | 0.461389 | 0.247312 | 0.024145298 | 2.533128435 |
| *TRRAP* | 0.482341 | 0.366667 | 0.061568189 | 2.183667368 |
| *FAT3* | 0.484128 | 0.434483 | 0.093370075 | 2.021796244 |
| *ATM* | 0.484128 | 0.434483 | 0.093370075 | 2.021796244 |
| *NF1* | 0.485537 | 3.571429 | 0.373913675 | 34.11242461 |
| *FANCI* | 0.485537 | 3.571429 | 0.373913675 | 34.11242461 |
| *INPP4A* | 0.485537 | 3.571429 | 0.373913675 | 34.11242461 |
| *PAK1* | 0.496673 | 0.535714 | 0.419798531 | 0.68363697 |
| *ADGRA2* | 0.496673 | 0.535714 | 0.419798531 | 0.68363697 |
| *BRAF* | 0.496673 | 0.535714 | 0.419798531 | 0.68363697 |
| *CCND2* | 0.496673 | 0.535714 | 0.419798531 | 0.68363697 |
| *NEB* | 0.496673 | 0.535714 | 0.419798531 | 0.68363697 |
| *NOTCH1* | 0.496673 | 0.535714 | 0.419798531 | 0.68363697 |
| *PIK3CG* | 0.496673 | 0.535714 | 0.419798531 | 0.68363697 |
| *STAT5B* | 0.496673 | 0.535714 | 0.419798531 | 0.68363697 |
| *TMPRSS2* | 0.496673 | 0.535714 | 0.419798531 | 0.68363697 |
| *BCL6* | 0.496673 | 0.535714 | 0.419798531 | 0.68363697 |
| *CASP8* | 0.496673 | 0.535714 | 0.419798531 | 0.68363697 |
| *CDKN1C* | 0.496673 | 0.535714 | 0.419798531 | 0.68363697 |
| *CHD2* | 0.496673 | 0.535714 | 0.419798531 | 0.68363697 |
| *EPHA7* | 0.496673 | 0.535714 | 0.419798531 | 0.68363697 |
| *FLT1* | 0.496673 | 0.535714 | 0.419798531 | 0.68363697 |
| *GNAS* | 0.496673 | 0.535714 | 0.419798531 | 0.68363697 |
| *HNF1B* | 0.496673 | 0.535714 | 0.419798531 | 0.68363697 |
| *INHBA* | 0.496673 | 0.535714 | 0.419798531 | 0.68363697 |
| *INPP4B* | 0.496673 | 0.535714 | 0.419798531 | 0.68363697 |
| *MAP3K1* | 0.496673 | 0.535714 | 0.419798531 | 0.68363697 |
| *MPL* | 0.496673 | 0.535714 | 0.419798531 | 0.68363697 |
| *NFKBIA* | 0.496673 | 0.535714 | 0.419798531 | 0.68363697 |
| *PIK3C2B* | 0.496673 | 0.535714 | 0.419798531 | 0.68363697 |
| *RASA1* | 0.496673 | 0.535714 | 0.419798531 | 0.68363697 |
| *TRAF2* | 0.496673 | 0.535714 | 0.419798531 | 0.68363697 |
| *ATR* | 0.605132 | 2.222222 | 0.39414097 | 12.52920144 |
| *NSD1* | 0.605132 | 2.222222 | 0.39414097 | 12.52920144 |
| *RBM10* | 0.605132 | 2.222222 | 0.39414097 | 12.52920144 |
| *SF3B1* | 0.605132 | 2.222222 | 0.39414097 | 12.52920144 |
| *KDM6A* | 0.635099 | 0.777778 | 0.275280027 | 2.197537825 |
| *CIC* | 0.760059 | 2.586207 | 0.252755431 | 26.46220537 |
| *PBRM1* | 0.760059 | 2.586207 | 0.252755431 | 26.46220537 |
| *ASXL1* | 0.760059 | 2.586207 | 0.252755431 | 26.46220537 |
| *SMARCA4* | 0.807782 | 0.511111 | 0.078787287 | 3.315694429 |
| *BRCA2* | 0.807782 | 0.511111 | 0.078787287 | 3.315694429 |
| *APC* | 0.807782 | 0.511111 | 0.078787287 | 3.315694429 |
| *CHEK2* | 0.807782 | 0.511111 | 0.078787287 | 3.315694429 |
| *TBX3* | 0.853235 | 0.387097 | 0.033108142 | 4.525893144 |
| *PTCH1* | 0.853235 | 0.387097 | 0.033108142 | 4.525893144 |
| *DOT1L* | 0.853235 | 0.387097 | 0.033108142 | 4.525893144 |
| *JUN* | 0.853235 | 0.387097 | 0.033108142 | 4.525893144 |
| *PMS2* | 0.853235 | 0.387097 | 0.033108142 | 4.525893144 |
| *RAD51D* | 0.853235 | 0.387097 | 0.033108142 | 4.525893144 |
| *EPHA5* | 0.853235 | 0.387097 | 0.033108142 | 4.525893144 |
| *FLT3* | 0.853235 | 0.387097 | 0.033108142 | 4.525893144 |
| *GATA3* | 0.853235 | 0.387097 | 0.033108142 | 4.525893144 |
| *LATS1* | 0.853235 | 0.387097 | 0.033108142 | 4.525893144 |
| *PTPRS* | 0.853235 | 0.387097 | 0.033108142 | 4.525893144 |
| *SPTA1* | 0.853235 | 0.387097 | 0.033108142 | 4.525893144 |
| *PIK3CA* | 0.867891 | 0.904762 | 0.278154935 | 2.942942943 |
| *FAT1* | 0.869397 | 1.714286 | 0.288287737 | 10.19389706 |
| *KMT2D* | 0.912165 | 1.060606 | 0.372810243 | 3.017313062 |
| *BAP1* | 0.947372 | 1.419753 | 0.305709716 | 6.593505923 |
| *KMT2A* | 0.947372 | 1.419753 | 0.305709716 | 6.593505923 |
| *KMT2C* | 0.984718 | 0.989418 | 0.33314044 | 2.93854435 |
| *ERCC1* | 1 | 0.54386 | 0.428779768 | 0.689825734 |
| *PGR* | 1 | 0.54386 | 0.428779768 | 0.689825734 |
| *FGFR1* | 1 | 0.54386 | 0.428779768 | 0.689825734 |
| *IGF1R* | 1 | 0.54386 | 0.428779768 | 0.689825734 |
| *INSR* | 1 | 0.54386 | 0.428779768 | 0.689825734 |
| *KRAS* | 1 | 0.54386 | 0.428779768 | 0.689825734 |
| *MET* | 1 | 0.54386 | 0.428779768 | 0.689825734 |
| *MLH1* | 1 | 0.54386 | 0.428779768 | 0.689825734 |
| *RAF1* | 1 | 0.54386 | 0.428779768 | 0.689825734 |
| *TNFRSF14* | 1 | 0.54386 | 0.428779768 | 0.689825734 |
| *CDKN1B* | 1 | 0.54386 | 0.428779768 | 0.689825734 |
| *CUL3* | 1 | 0.54386 | 0.428779768 | 0.689825734 |
| *EPHB1* | 1 | 0.54386 | 0.428779768 | 0.689825734 |
| *ERRFI1* | 1 | 0.54386 | 0.428779768 | 0.689825734 |
| *GALNT12* | 1 | 0.54386 | 0.428779768 | 0.689825734 |
| *HSP90AA1* | 1 | 0.54386 | 0.428779768 | 0.689825734 |
| *IKZF1* | 1 | 0.54386 | 0.428779768 | 0.689825734 |
| *KEAP1* | 1 | 0.54386 | 0.428779768 | 0.689825734 |
| *NOTCH3* | 1 | 0.54386 | 0.428779768 | 0.689825734 |
| *NTRK2* | 1 | 0.54386 | 0.428779768 | 0.689825734 |
| *PPP6C* | 1 | 0.54386 | 0.428779768 | 0.689825734 |
| *RAD52* | 1 | 0.54386 | 0.428779768 | 0.689825734 |
| *AMER1* | 1 | 0.54386 | 0.428779768 | 0.689825734 |
| *AR* | 1 | 0.54386 | 0.428779768 | 0.689825734 |
| *AURKB* | 1 | 0.54386 | 0.428779768 | 0.689825734 |
| *CCND3* | 1 | 0.54386 | 0.428779768 | 0.689825734 |
| *CCNE1* | 1 | 0.54386 | 0.428779768 | 0.689825734 |
| *CHD1* | 1 | 0.54386 | 0.428779768 | 0.689825734 |
| *CHEK1* | 1 | 0.54386 | 0.428779768 | 0.689825734 |
| *DIS3* | 1 | 0.54386 | 0.428779768 | 0.689825734 |
| *EPHA2* | 1 | 0.54386 | 0.428779768 | 0.689825734 |
| *FGF12* | 1 | 0.54386 | 0.428779768 | 0.689825734 |
| *FOXP1* | 1 | 0.54386 | 0.428779768 | 0.689825734 |
| *GNA11* | 1 | 0.54386 | 0.428779768 | 0.689825734 |
| *H3F3A* | 1 | 0.54386 | 0.428779768 | 0.689825734 |
| *HIST1H3E* | 1 | 0.54386 | 0.428779768 | 0.689825734 |
| *IRS1* | 1 | 0.54386 | 0.428779768 | 0.689825734 |
| *KIT* | 1 | 0.54386 | 0.428779768 | 0.689825734 |
| *LATS2* | 1 | 0.54386 | 0.428779768 | 0.689825734 |
| *MAX* | 1 | 0.54386 | 0.428779768 | 0.689825734 |
| *MDM4* | 1 | 0.54386 | 0.428779768 | 0.689825734 |
| *MEF2B* | 1 | 0.54386 | 0.428779768 | 0.689825734 |
| *MSH2* | 1 | 0.54386 | 0.428779768 | 0.689825734 |
| *MUTYH* | 1 | 0.54386 | 0.428779768 | 0.689825734 |
| *NCOA3* | 1 | 0.54386 | 0.428779768 | 0.689825734 |
| *NFE2L2* | 1 | 0.54386 | 0.428779768 | 0.689825734 |
| *NKX2_1* | 1 | 0.54386 | 0.428779768 | 0.689825734 |
| *NOTCH4* | 1 | 0.54386 | 0.428779768 | 0.689825734 |
| *PAK5* | 1 | 0.54386 | 0.428779768 | 0.689825734 |
| *PARP4* | 1 | 0.54386 | 0.428779768 | 0.689825734 |
| *PIK3C3* | 1 | 0.54386 | 0.428779768 | 0.689825734 |
| *PIK3CB* | 1 | 0.54386 | 0.428779768 | 0.689825734 |
| *POM121L12* | 1 | 0.54386 | 0.428779768 | 0.689825734 |
| *PTK2* | 1 | 0.54386 | 0.428779768 | 0.689825734 |
| *RECQL4* | 1 | 0.54386 | 0.428779768 | 0.689825734 |
| *RET* | 1 | 0.54386 | 0.428779768 | 0.689825734 |
| *SDHAF2* | 1 | 0.54386 | 0.428779768 | 0.689825734 |
| *SDHB* | 1 | 0.54386 | 0.428779768 | 0.689825734 |
| *SLX4* | 1 | 0.54386 | 0.428779768 | 0.689825734 |
| *SMARCB1* | 1 | 0.54386 | 0.428779768 | 0.689825734 |
| *SOX10* | 1 | 0.54386 | 0.428779768 | 0.689825734 |
| *TACC3* | 1 | 0.54386 | 0.428779768 | 0.689825734 |
| *TAF1* | 1 | 0.54386 | 0.428779768 | 0.689825734 |
| *TCF3* | 1 | 0.54386 | 0.428779768 | 0.689825734 |
| *TENT5C* | 1 | 0.54386 | 0.428779768 | 0.689825734 |
| *TGFBR2* | 1 | 0.54386 | 0.428779768 | 0.689825734 |
| *TP63* | 1 | 0.54386 | 0.428779768 | 0.689825734 |
| *VEGFA* | 1 | 0.54386 | 0.428779768 | 0.689825734 |
| *XPO1* | 1 | 0.54386 | 0.428779768 | 0.689825734 |
| *ZNF703* | 1 | 0.54386 | 0.428779768 | 0.689825734 |
| *ZNRF3* | 1 | 0.54386 | 0.428779768 | 0.689825734 |
| *FBXW7* | 1 | 0.793103 | 0.146154638 | 4.303750396 |
| *NBN* | 1 | 0.793103 | 0.146154638 | 4.303750396 |
| *CTNNB1* | 1 | 0.793103 | 0.146154638 | 4.303750396 |
| *PMS1* | 1 | 0.793103 | 0.146154638 | 4.303750396 |
| *CDK12* | 1 | 1.241379 | 0.191487656 | 8.047634111 |
| *ASXL2* | 1 | 0.8 | 0.104844449 | 6.104281192 |
| *BLM* | 1 | 0.8 | 0.104844449 | 6.104281192 |
| *JAK3* | 1 | 0.8 | 0.104844449 | 6.104281192 |
| *RAD51C* | 1 | 0.8 | 0.104844449 | 6.104281192 |
| *CDKN1A* | 1 | 0.8 | 0.104844449 | 6.104281192 |
| *MLH3* | 1 | 0.8 | 0.104844449 | 6.104281192 |
| *ZNF217* | 1 | 0.8 | 0.104844449 | 6.104281192 |
| *DAXX* | 1 | 1.666667 | 0.142614818 | 19.4774836 |
| *EGFR* | 1 | 1.666667 | 0.142614818 | 19.4774836 |
| *SMO* | 1 | 1.666667 | 0.142614818 | 19.4774836 |
| *CHD4* | 1 | 1.666667 | 0.142614818 | 19.4774836 |
| *JAK2* | 1 | 1.666667 | 0.142614818 | 19.4774836 |
| *KDR* | 1 | 1.666667 | 0.142614818 | 19.4774836 |
| *MST1R* | 1 | 1.666667 | 0.142614818 | 19.4774836 |
| *NCOR1* | 1 | 1.666667 | 0.142614818 | 19.4774836 |
| *PREX2* | 1 | 1.666667 | 0.142614818 | 19.4774836 |
| *RPTOR* | 1 | 1.666667 | 0.142614818 | 19.4774836 |
| *FOXA1* | 1 | 1.666667 | 0.142614818 | 19.4774836 |
| *GNA13* | 1 | 1.666667 | 0.142614818 | 19.4774836 |
| *MCL1* | 1 | 1.666667 | 0.142614818 | 19.4774836 |
| *MTOR* | 1 | 1.666667 | 0.142614818 | 19.4774836 |
| *PTEN* | 1 | 1.666667 | 0.142614818 | 19.4774836 |
| *RB1* | 1 | 1.666667 | 0.142614818 | 19.4774836 |
| *RICTOR* | 1 | 1.666667 | 0.142614818 | 19.4774836 |
| *PLCG2* | 1 | 0.806452 | 0.048000391 | 13.54914391 |
| *ROS1* | 1 | 0.806452 | 0.048000391 | 13.54914391 |
| *BRCA1* | 1 | 0.806452 | 0.048000391 | 13.54914391 |
| *CUL4B* | 1 | 0.806452 | 0.048000391 | 13.54914391 |
| *EWSR1* | 1 | 0.806452 | 0.048000391 | 13.54914391 |
| *GLI1* | 1 | 0.806452 | 0.048000391 | 13.54914391 |
| *HIST1H1C* | 1 | 0.806452 | 0.048000391 | 13.54914391 |
| *IRS2* | 1 | 0.806452 | 0.048000391 | 13.54914391 |
| *PARP3* | 1 | 0.806452 | 0.048000391 | 13.54914391 |
| *RNF43* | 1 | 0.806452 | 0.048000391 | 13.54914391 |
| *RUNX1* | 1 | 0.806452 | 0.048000391 | 13.54914391 |
| *SMAD2* | 1 | 0.806452 | 0.048000391 | 13.54914391 |
| *SYK* | 1 | 0.806452 | 0.048000391 | 13.54914391 |
| *TET1* | 1 | 0.806452 | 0.048000391 | 13.54914391 |
| *TET2* | 1 | 0.806452 | 0.048000391 | 13.54914391 |
| *ABL1* | 1 | 0.806452 | 0.048000391 | 13.54914391 |
| *ALOX12B* | 1 | 0.806452 | 0.048000391 | 13.54914391 |
| *BCL2L1* | 1 | 0.806452 | 0.048000391 | 13.54914391 |
| *BRD4* | 1 | 0.806452 | 0.048000391 | 13.54914391 |
| *DNMT3B* | 1 | 0.806452 | 0.048000391 | 13.54914391 |
| *FH* | 1 | 0.806452 | 0.048000391 | 13.54914391 |
| *FUBP1* | 1 | 0.806452 | 0.048000391 | 13.54914391 |
| *GATA2* | 1 | 0.806452 | 0.048000391 | 13.54914391 |
| *HIST1H3I* | 1 | 0.806452 | 0.048000391 | 13.54914391 |
| *KDM5C* | 1 | 0.806452 | 0.048000391 | 13.54914391 |
| *MED12* | 1 | 0.806452 | 0.048000391 | 13.54914391 |
| *MYC* | 1 | 0.806452 | 0.048000391 | 13.54914391 |
| *RANBP2* | 1 | 0.806452 | 0.048000391 | 13.54914391 |
| *SOX2* | 1 | 0.806452 | 0.048000391 | 13.54914391 |
| *CCND1* | NA | NA | NA | NA |
| *FGF19* | NA | NA | NA | NA |
| *FGF4* | NA | NA | NA | NA |
| *CDKN2A* | NA | NA | NA | NA |
| *CDKN2B* | NA | NA | NA | NA |
| *MDM2* | NA | NA | NA | NA |
| *HNF1A* | NA | NA | NA | NA |
| *TOP1* | NA | NA | NA | NA |
| *TSC2* | NA | NA | NA | NA |
| *ACVR1B* | NA | NA | NA | NA |
| *AXIN1* | NA | NA | NA | NA |
| *BACH1* | NA | NA | NA | NA |
| *CD79A* | NA | NA | NA | NA |
| *CD79B* | NA | NA | NA | NA |
| *CDK4* | NA | NA | NA | NA |
| *CDK6* | NA | NA | NA | NA |
| *CYLD* | NA | NA | NA | NA |
| *EPHA3* | NA | NA | NA | NA |
| *FANCL* | NA | NA | NA | NA |
| *FGFR2* | NA | NA | NA | NA |
| *GREM1* | NA | NA | NA | NA |
| *HLA_A* | NA | NA | NA | NA |
| *MAP2K2* | NA | NA | NA | NA |
| *NF2* | NA | NA | NA | NA |
| *NKX3_1* | NA | NA | NA | NA |
| *NR4A3* | NA | NA | NA | NA |
| *NTHL1* | NA | NA | NA | NA |
| *NUP93* | NA | NA | NA | NA |
| *PDGFRA* | NA | NA | NA | NA |
| *PIK3CD* | NA | NA | NA | NA |
| *PRDM1* | NA | NA | NA | NA |
| *PRKN* | NA | NA | NA | NA |
| *RAC1* | NA | NA | NA | NA |
| *RARA* | NA | NA | NA | NA |
| *SOX9* | NA | NA | NA | NA |
| *SRC* | NA | NA | NA | NA |
| *STAT3* | NA | NA | NA | NA |

CI: confidence interval; NA: not available.

**Table S3.** Correlation between single genetic alterations and prediction models

| Gene | P value | |
| --- | --- | --- |
|  | **CUETO score** | **EORTC score** |
| *KDM6A* | 0.863 | 0.038 |
| *KMT2D* | 0.184 | 0.557 |
| *FGFR3* | 0.164 | 0.210 |
| *KMT2C* | 0.870 | 0.540 |
| *STAG2* | 0.793 | 0.639 |
| *CREBBP* | 0.275 | 0.086 |
| *PIK3CA* | 0.821 | 0.353 |
| *TP53* | 0.018 | 0.915 |
| *ARID1A* | 0.109 | 0.004 |
| *TERT* | 0.216 | 0.941 |
| *ERBB2* | 0.487 | 0.858 |
| *FANCA* | 0.416 | 0.995 |
| *FAT3* | 0.744 | 0.002 |
| *ATM* | 0.503 | 0.856 |
| *BAP1* | 0.340 | 0.647 |
| *LRP1B* | 0.601 | 0.622 |
| *KMT2A* | 0.632 | 0.693 |
| *ATR* | 0.847 | 0.453 |
| *NSD1* | 0.350 | 0.182 |
| *RBM10* | 0.988 | 0.064 |
| *SF3B1* | 0.247 | 0.208 |
| *TSC1* | 0.876 | 0.447 |
| *FBXW7* | 0.828 | 0.117 |
| *FAT1* | 0.148 | 0.167 |
| *NBN* | 0.612 | 0.425 |
| *PRKDC* | 0.487 | 0.683 |
| *TRRAP* | 0.890 | 0.149 |
| *U2AF1* | 0.868 | 0.997 |
| *CTNNB1* | 0.805 | 0.455 |
| *PMS1* | 0.176 | 0.032 |
| *EP300* | 0.039 | 0.262 |
| *SMARCA4* | 0.936 | 0.555 |
| *ARID1B* | 0.616 | 0.523 |
| *BRCA2* | 0.733 | 0.016 |
| *ERBB3* | 0.733 | 0.949 |
| *NF1* | 0.616 | 0.555 |
| *APC* | 0.896 | 0.193 |
| *BRIP1* | 0.159 | 0.042 |
| *CDK12* | 0.896 | 0.555 |
| *FANCI* | 0.444 | 0.443 |
| *CHEK2* | 0.580 | 0.678 |
| *HRAS* | 0.444 | 0.443 |
| *INPP4A* | 0.936 | 0.555 |
| *ASXL2* | 0.658 | 0.799 |
| *BLM* | 0.658 | 0.360 |
| *CIC* | 0.103 | 0.034 |
| *JAK3* | 0.498 | 0.601 |
| *PBRM1* | 0.834 | 0.952 |
| *RAD51C* | 0.361 | 0.034 |
| *SETD2* | 0.361 | 0.131 |
| *AKT1* | 0.834 | 0.601 |
| *ARID2* | 0.250 | 0.056 |
| *ASXL1* | 0.468 | 0.799 |
| *CDKN1A* | 0.250 | 0.519 |
| *CSF1R* | 0.468 | 0.519 |
| *MLH3* | 0.361 | 0.190 |
| *ZNF217* | 0.797 | 0.131 |
| *TBX3* | 0.963 | 0.041 |
| *ERCC2* | 0.395 | 0.115 |
| *SDHC* | 0.449 | 0.821 |
| *ERBB4* | 0.064 | 0.659 |
| *PTCH1* | 0.262 | 0.012 |
| *DAXX* | 0.262 | 0.827 |
| *DOT1L* | 0.561 | 0.654 |
| *EGFR* | 0.754 | 0.262 |
| *JUN* | 0.095 | 0.502 |
| *SMO* | 0.625 | 0.654 |
| *ATRX* | 0.625 | 0.821 |
| *CHD4* | 0.754 | 0.177 |
| *FLT4* | 0.449 | 0.827 |
| *JAK2* | 0.963 | 0.821 |
| *KDR* | 0.561 | 0.997 |
| *MST1R* | 0.963 | 0.115 |
| *NCOR1* | 0.164 | 0.262 |
| *PMS2* | 0.754 | 0.502 |
| *PREX2* | 0.051 | 0.654 |
| *PTPRD* | 0.625 | 0.506 |
| *RAD51D* | 0.625 | 0.821 |
| *RPTOR* | 0.561 | 0.827 |
| *ABL2* | 0.116 | 0.117 |
| *BCORL1* | 0.164 | 0.370 |
| *EPHA5* | 0.754 | 0.071 |
| *FLT3* | 0.304 | 0.370 |
| *FOXA1* | 0.754 | 0.177 |
| *GATA3* | 0.754 | 0.370 |
| *GNA13* | 0.262 | 0.654 |
| *IDH1* | 0.963 | 0.262 |
| *JAK1* | 0.304 | 0.042 |
| *LATS1* | 0.825 | 0.071 |
| *MCL1* | 0.625 | 0.827 |
| *MSH3* | 0.304 | 0.502 |
| *MTOR* | 0.116 | 0.659 |
| *POLE* | 0.963 | 0.827 |
| *PTEN* | 0.625 | 0.374 |
| *PTPRS* | 0.561 | 0.821 |
| *RAD50* | 0.304 | 0.117 |
| *RAD54L* | 0.164 | 0.177 |
| *RB1* | 0.304 | 0.072 |
| *RICTOR* | 0.963 | 0.827 |
| *SPEN* | 0.825 | 0.370 |
| *SPTA1* | 0.064 | 0.654 |
| *DDR2* | 0.858 | 0.473 |
| *PLCG2* | 0.407 | 0.860 |
| *PTPN11* | 0.884 | 0.044 |
| *PAK1* | 0.426 | 0.717 |
| *ROS1* | 0.407 | 0.926 |
| *ADGRA2* | 0.858 | 0.926 |
| *BRAF* | 0.638 | 0.860 |
| *BRCA1* | 0.884 | 0.083 |
| *CCND2* | 0.858 | 0.717 |
| *CTCF* | 0.426 | 0.238 |
| *CUL4B* | 0.068 | 0.365 |
| *EWSR1* | 0.638 | 0.083 |
| *GLI1* | 0.615 | 0.717 |
| *HIST1H1C* | 0.426 | 0.365 |
| *IRS2* | 0.638 | 0.145 |
| *NEB* | 0.136 | 0.017 |
| *NOTCH1* | 0.615 | 0.473 |
| *PARP3* | 0.858 | 0.238 |
| *PIK3CG* | 0.615 | 0.860 |
| *PTPRT* | 0.426 | 0.145 |
| *RNF43* | 0.426 | 0.365 |
| *RUNX1* | 0.426 | 0.655 |
| *SMAD2* | 0.426 | 0.238 |
| *STAT5B* | 0.261 | 0.365 |
| *SYK* | 0.884 | 0.655 |
| *TET1* | 0.615 | 0.860 |
| *TET2* | 0.426 | 0.365 |
| *TMPRSS2* | 0.884 | 0.238 |
| *WT1* | 0.247 | 0.655 |
| *ABL1* | 0.884 | 0.860 |
| *ALOX12B* | 0.615 | 0.526 |
| *BCL2L1* | 0.426 | 0.526 |
| *BCL6* | 0.407 | 0.860 |
| *BCOR* | 0.247 | 0.655 |
| *BRD4* | 0.638 | 0.365 |
| *CASP8* | 0.858 | 0.717 |
| *CDKN1C* | 0.247 | 0.717 |
| *CHD2* | 0.615 | 0.206 |
| *DNMT3B* | 0.615 | 0.365 |
| *EPHA7* | 0.858 | 0.717 |
| *FH* | 0.638 | 0.238 |
| *FLT1* | 0.030 | 0.206 |
| *FUBP1* | 0.884 | 0.655 |
| *GATA2* | 0.146 | 0.365 |
| *GNAS* | 0.615 | 0.717 |
| *HIST1H3I* | 0.884 | 0.926 |
| *HNF1B* | 0.638 | 0.526 |
| *INHBA* | 0.858 | 0.926 |
| *INPP4B* | 0.068 | 0.473 |
| *KDM5C* | 0.858 | 0.238 |
| *MAP3K1* | 0.407 | 0.365 |
| *MED12* | 0.638 | 0.365 |
| *MPL* | 0.068 | 0.717 |
| *MYC* | 0.884 | 0.526 |
| *NFKBIA* | 0.426 | 0.526 |
| *PALB2* | 0.884 | 0.926 |
| *PIK3C2B* | 0.068 | 0.322 |
| *PIK3C2G* | 0.638 | 0.717 |
| *RANBP2* | 0.884 | 0.526 |
| *RASA1* | 0.261 | 0.238 |
| *SOX2* | 0.615 | 0.145 |
| *TRAF2* | 0.615 | 0.926 |
| *FGF3* | 0.742 | 0.310 |
| *ERCC1* | 0.432 | 0.310 |
| *SH2B3* | 0.742 | 0.161 |
| *PGR* | 0.213 | 0.310 |
| *EMSY* | 0.432 | 0.799 |
| *FANCD2* | 0.742 | 0.073 |
| *FGFR1* | 0.133 | 0.004 |
| *IGF1R* | 0.742 | 0.799 |
| *INSR* | 0.299 | 0.526 |
| *KDM5A* | 0.742 | 0.615 |
| *KRAS* | 0.086 | 0.799 |
| *MET* | 0.299 | 0.526 |
| *MLH1* | 0.562 | 0.901 |
| *MRE11* | 0.742 | 0.073 |
| *RAF1* | 0.562 | 0.615 |
| *RUNX1T1* | 0.742 | 0.526 |
| *TNFAIP3* | 0.900 | 0.310 |
| *TNFRSF14* | 0.432 | 0.799 |
| *ALK* | 0.742 | 0.073 |
| *ANKRD11* | 0.742 | 0.073 |
| *CARD11* | 0.742 | 0.073 |
| *CDKN1B* | 0.562 | 0.901 |
| *CRLF2* | 0.432 | 0.526 |
| *CUL3* | 0.900 | 0.526 |
| *EPHB1* | 0.742 | 0.205 |
| *ERRFI1* | 0.299 | 0.615 |
| *GALNT12* | 0.299 | 0.615 |
| *GRIN2A* | 0.742 | 0.310 |
| *HSP90AA1* | 0.742 | 0.615 |
| *IKZF1* | 0.432 | 0.799 |
| *KEAP1* | 0.213 | 0.310 |
| *MST1* | 0.213 | 0.799 |
| *NOTCH2* | 0.432 | 0.526 |
| *NOTCH3* | 0.432 | 0.310 |
| *NTRK1* | 0.742 | 0.161 |
| *NTRK2* | 0.742 | 0.615 |
| *PDGFRB* | 0.742 | 0.526 |
| *PIK3R2* | 0.432 | 0.799 |
| *PPP2R1A* | 0.213 | 0.377 |
| *PPP6C* | 0.900 | 0.526 |
| *PRKAR1A* | 0.742 | 0.526 |
| *RAD51* | 0.432 | 0.799 |
| *RAD52* | 0.562 | 0.377 |
| *TIPARP* | 0.900 | 0.310 |
| *ZFHX3* | 0.432 | 0.799 |
| *AMER1* | 0.562 | 0.615 |
| *AR* | 0.742 | 0.310 |
| *ARID5B* | 0.742 | 0.073 |
| *AURKB* | 0.742 | 0.526 |
| *B2M* | 0.432 | 0.799 |
| *BCL10* | 0.742 | 0.073 |
| *CCND3* | 0.432 | 0.310 |
| *CCNE1* | 0.562 | 0.377 |
| *CEBPA* | 0.742 | 0.161 |
| *CHD1* | 0.432 | 0.799 |
| *CHEK1* | 0.133 | 0.040 |
| *CSF3R* | 0.742 | 0.377 |
| *CXCR4* | 0.432 | 0.799 |
| *DIS3* | 0.432 | 0.799 |
| *DNAJB1* | 0.432 | 0.799 |
| *EPHA2* | 0.742 | 0.799 |
| *ERCC4* | 0.213 | 0.799 |
| *FANCE* | 0.432 | 0.799 |
| *FANCF* | 0.432 | 0.799 |
| *FGF12* | 0.003 | 0.615 |
| *FGFR4* | 0.900 | 0.310 |
| *FOXP1* | 0.432 | 0.799 |
| *GNA11* | 0.742 | 0.310 |
| *GNAQ* | 0.432 | 0.799 |
| *H3F3A* | 0.213 | 0.310 |
| *H3F3C* | 0.900 | 0.310 |
| *HGF* | 0.432 | 0.799 |
| *HIST1H3E* | 0.562 | 0.901 |
| *HIST1H3J* | 0.742 | 0.377 |
| *IGF2* | 0.213 | 0.377 |
| *IRS1* | 0.742 | 0.799 |
| *KAT6A* | 0.432 | 0.799 |
| *KIT* | 0.432 | 0.901 |
| *LATS2* | 0.432 | 0.901 |
| *MAGI2* | 0.028 | 0.161 |
| *MAP3K13* | 0.742 | 0.526 |
| *MAX* | 0.133 | 0.004 |
| *MDM4* | 0.742 | 0.526 |
| *MEF2B* | 0.900 | 0.526 |
| *MSH2* | 0.742 | 0.526 |
| *MSH6* | 0.900 | 0.310 |
| *MUTYH* | 0.432 | 0.901 |
| *NCOA3* | 0.133 | 0.799 |
| *NFE2L2* | 0.432 | 0.901 |
| *NKX2_1* | 0.562 | 0.901 |
| *NOTCH4* | 0.562 | 0.901 |
| *NRAS* | 0.432 | 0.526 |
| *NTRK3* | 0.742 | 0.073 |
| *PAK5* | 0.133 | 0.040 |
| *PARP4* | 0.742 | 0.526 |
| *PAX5* | 0.742 | 0.073 |
| *PIK3C3* | 0.742 | 0.205 |
| *PIK3CB* | 0.742 | 0.615 |
| *POLD1* | 0.432 | 0.799 |
| *POM121L12* | 0.213 | 0.310 |
| *PTK2* | 0.133 | 0.799 |
| *RECQL4* | 0.562 | 0.615 |
| *RET* | 0.562 | 0.901 |
| *RHOA* | 0.742 | 0.615 |
| *RIT1* | 0.742 | 0.073 |
| *SDHAF2* | 0.562 | 0.615 |
| *SDHB* | 0.562 | 0.901 |
| *SLX4* | 0.432 | 0.799 |
| *SMARCB1* | 0.213 | 0.310 |
| *SMARCD1* | 0.432 | 0.526 |
| *SOX10* | 0.133 | 0.799 |
| *TACC3* | 0.742 | 0.615 |
| *TAF1* | 0.003 | 0.615 |
| *TCF3* | 0.299 | 0.901 |
| *TENT5C* | 0.133 | 0.040 |
| *TGFBR2* | 0.742 | 0.310 |
| *TNFSF11* | 0.900 | 0.310 |
| *TP63* | 0.133 | 0.799 |
| *VEGFA* | 0.742 | 0.310 |
| *WRN* | 0.432 | 0.799 |
| *XIAP* | 0.742 | 0.073 |
| *XPO1* | 0.742 | 0.310 |
| *ZNF703* | 0.742 | 0.205 |
| *ZNRF3* | 0.562 | 0.901 |
| *CCND1* | 0.000 | 0.000 |
| *FGF19* | NA | NA |
| *FGF4* | NA | NA |
| *CDKN2A* | NA | NA |
| *CDKN2B* | NA | NA |
| *MDM2* | NA | NA |
| *HNF1A* | NA | NA |
| *TOP1* | NA | NA |
| *TSC2* | NA | NA |
| *ACVR1B* | NA | NA |
| *AXIN1* | NA | NA |
| *BACH1* | NA | NA |
| *CD79A* | NA | NA |
| *CD79B* | NA | NA |
| *CDK4* | NA | NA |
| *CDK6* | NA | NA |
| *CYLD* | NA | NA |
| *EPHA3* | NA | NA |
| *FANCL* | NA | NA |
| *FGFR2* | NA | NA |
| *GREM1* | NA | NA |
| *HLA_A* | NA | NA |
| *MAP2K2* | NA | NA |
| *NF2* | NA | NA |
| *NKX3_1* | NA | NA |
| *NR4A3* | NA | NA |
| *NTHL1* | NA | NA |
| *NUP93* | NA | NA |
| *PDGFRA* | NA | NA |
| *PIK3CD* | NA | NA |
| *PRDM1* | NA | NA |
| *PRKN* | NA | NA |
| *RAC1* | NA | NA |
| *RARA* | NA | NA |
| *SOX9* | NA | NA |
| *SRC* | NA | NA |
| *STAT3* | NA | NA |
| *STK40* | NA | NA |
| *SUFU* | NA | NA |
| *ZBTB2* | NA | NA |

Only those genes mutated in more than 10% of patients and those significantly correlated with RFS were analyzed.

**Table S4.** Univariate analysis of total NMIBC cohort (n=58)

| Gene | P | Hazard ratio | Lower limit of 95% CI | Upper limit of 95% CI |
| --- | --- | --- | --- | --- |
| *KDM6A* | 0.076 | 0.359 | 0.116 | 1.114 |
| *KMT2D* | 0.394 | 1.539 | 0.571 | 4.149 |
| *FGFR3* | 0.611 | 1.290 | 0.483 | 3.445 |
| *KMT2C* | 0.213 | 1.865 | 0.699 | 4.979 |
| *STAG2* | 0.178 | 1.992 | 0.731 | 5.431 |
| *CREBBP* | 0.142 | 0.328 | 0.074 | 1.450 |
| *PIK3CA* | 0.929 | 0.949 | 0.304 | 2.968 |
| *TP53* | 0.936 | 1.048 | 0.336 | 3.266 |
| *ARID1A* | 0.165 | 0.346 | 0.078 | 1.546 |
| *TERT* | 0.406 | 1.618 | 0.521 | 5.030 |
| *ERBB2* | 0.692 | 0.741 | 0.168 | 3.263 |
| *FANCA* | 0.569 | 1.442 | 0.410 | 5.078 |
| *FAT3* | 0.264 | 0.038 | 0.000 | 11.838 |
| *ATM* | 0.915 | 0.922 | 0.209 | 4.064 |
| *BAP1* | 0.267 | 0.038 | 0.000 | 12.261 |
| *LRP1B* | 0.362 | 0.390 | 0.051 | 2.953 |
| *KMT2A* | 0.942 | 1.056 | 0.240 | 4.656 |
| *ATR* | 0.502 | 0.499 | 0.066 | 3.798 |
| *NSD1* | 0.335 | 1.866 | 0.525 | 6.635 |
| *RBM10* | 0.307 | 0.039 | 0.000 | 19.554 |
| *SF3B1* | 0.454 | 0.462 | 0.061 | 3.501 |
| *TSC1* | 0.370 | 0.393 | 0.051 | 3.030 |
| *FBXW7* | 0.536 | 1.599 | 0.362 | 7.061 |
| *FAT1* | 0.653 | 1.406 | 0.318 | 6.213 |
| *NBN* | 0.508 | 0.503 | 0.066 | 3.850 |
| *PRKDC* | 0.182 | 2.355 | 0.669 | 8.293 |
| *TRRAP* | 0.552 | 0.541 | 0.071 | 4.100 |
| *U2AF1* | 0.673 | 0.646 | 0.085 | 4.924 |
| *CTNNB1* | 0.638 | 1.428 | 0.324 | 6.288 |
| *PMS1* | 0.566 | 0.552 | 0.073 | 4.187 |
| *EP300* | 0.571 | 0.556 | 0.073 | 4.225 |
| *SMARCA4* | 0.667 | 0.641 | 0.085 | 4.855 |
| *ARID1B* | 0.717 | 0.688 | 0.091 | 5.208 |
| *BRCA2* | 0.411 | 0.043 | 0.000 | 78.034 |
| *ERBB3* | 0.385 | 0.042 | 0.000 | 53.217 |
| *NF1* | 0.637 | 0.613 | 0.081 | 4.663 |
| *APC* | 0.377 | 1.964 | 0.439 | 8.798 |
| *BRIP1* | 0.701 | 0.673 | 0.089 | 5.097 |
| *CDK12* | 0.549 | 0.536 | 0.070 | 4.114 |
| *FANCI* | 0.449 | 1.778 | 0.401 | 7.890 |
| *CHEK2* | 0.667 | 0.641 | 0.085 | 4.855 |
| *HRAS* | 0.771 | 0.740 | 0.098 | 5.614 |
| *INPP4A* | 0.674 | 0.647 | 0.085 | 4.920 |
| *ASXL2* | 0.190 | 2.718 | 0.610 | 12.121 |
| *BLM* | 0.910 | 1.125 | 0.147 | 8.628 |
| *CIC* | 0.462 | 0.044 | 0.000 | 178.358 |
| *JAK3* | 0.986 | 1.018 | 0.132 | 7.861 |
| *PBRM1* | 0.437 | 0.044 | 0.000 | 116.845 |
| *RAD51C* | 0.424 | 0.041 | 0.000 | 102.939 |
| *SETD2* | 0.462 | 0.044 | 0.000 | 178.358 |
| *AKT1* | 0.999 | 1.002 | 0.131 | 7.631 |
| *ARID2* | 0.493 | 0.045 | 0.000 | 326.471 |
| *ASXL1* | 0.975 | 0.968 | 0.127 | 7.365 |
| *CDKN1A* | 0.844 | 0.816 | 0.107 | 6.241 |
| *CSF1R* | 0.234 | 2.469 | 0.557 | 10.938 |
| *MLH3* | 0.968 | 0.959 | 0.126 | 7.310 |
| *ZNF217* | 0.985 | 1.019 | 0.134 | 7.767 |
| *TBX3* | 0.634 | 1.642 | 0.214 | 12.603 |
| *ERCC2* | 0.520 | 0.045 | 0.000 | 558.925 |
| *SDHC* | 0.074 | 3.917 | 0.877 | 17.507 |
| *ERBB4* | 0.769 | 1.356 | 0.178 | 10.322 |
| *PTCH1* | 0.530 | 0.046 | 0.000 | 693.194 |
| *DAXX* | 0.799 | 1.301 | 0.171 | 9.911 |
| *DOT1L* | 0.520 | 0.045 | 0.000 | 558.925 |
| *EGFR* | 0.518 | 1.959 | 0.255 | 15.028 |
| *JUN* | 0.527 | 0.045 | 0.000 | 663.046 |
| *SMO* | 0.771 | 1.352 | 0.178 | 10.285 |
| *ATRX* | 0.176 | 2.797 | 0.630 | 12.421 |
| *CHD4* | 0.472 | 0.044 | 0.000 | 221.212 |
| *FLT4* | 0.968 | 1.042 | 0.137 | 7.912 |
| *JAK2* | 0.506 | 0.045 | 0.000 | 418.359 |
| *KDR* | 0.527 | 0.045 | 0.000 | 663.046 |
| *MST1R* | 0.477 | 2.098 | 0.272 | 16.197 |
| *NCOR1* | 0.492 | 0.045 | 0.000 | 314.150 |
| *PMS2* | 0.561 | 0.046 | 0.000 | 1484.195 |
| *PREX2* | 0.766 | 1.362 | 0.178 | 10.422 |
| *PTPRD* | 0.472 | 0.044 | 0.000 | 221.212 |
| *RAD51D* | 0.123 | 3.236 | 0.727 | 14.408 |
| *RPTOR* | 0.520 | 0.045 | 0.000 | 558.925 |
| *ABL2* | 0.942 | 1.078 | 0.139 | 8.336 |
| *BCORL1* | 0.499 | 0.045 | 0.000 | 360.842 |
| *EPHA5* | 0.492 | 0.045 | 0.000 | 314.150 |
| *FLT3* | 0.771 | 1.352 | 0.178 | 10.285 |
| *FOXA1* | 0.804 | 1.293 | 0.170 | 9.836 |
| *GATA3* | 0.024 | 6.140 | 1.263 | 29.846 |
| *GNA13* | 0.472 | 0.044 | 0.000 | 221.212 |
| *IDH1* | 0.520 | 0.045 | 0.000 | 558.925 |
| *JAK1* | 0.506 | 0.045 | 0.000 | 418.359 |
| *LATS1* | 0.499 | 0.045 | 0.000 | 360.842 |
| *MCL1* | 0.956 | 1.059 | 0.140 | 8.030 |
| *MSH3* | 0.673 | 1.548 | 0.203 | 11.790 |
| *MTOR* | 0.052 | 4.442 | 0.989 | 19.956 |
| *POLE* | 0.790 | 1.318 | 0.173 | 10.039 |
| *PTEN* | 0.472 | 0.044 | 0.000 | 221.212 |
| *PTPRS* | 0.735 | 1.420 | 0.186 | 10.813 |
| *RAD50* | 0.767 | 1.359 | 0.179 | 10.342 |
| *RAD54L* | 0.561 | 0.046 | 0.000 | 1484.195 |
| *RB1* | 0.749 | 1.394 | 0.183 | 10.615 |
| *RICTOR* | 0.506 | 0.045 | 0.000 | 418.359 |
| *SPEN* | 0.673 | 1.548 | 0.203 | 11.790 |
| *SPTA1* | 0.128 | 3.178 | 0.716 | 14.117 |
| *DDR2* | 0.389 | 2.451 | 0.319 | 18.822 |
| *PLCG2* | 0.568 | 0.046 | 0.000 | 1763.214 |
| *PTPN11* | 0.244 | 3.444 | 0.430 | 27.609 |
| *PAK1* | 0.593 | 1.742 | 0.228 | 13.335 |
| *ROS1* | 0.592 | 1.741 | 0.229 | 13.250 |
| *ADGRA2* | 0.614 | 0.047 | 0.000 | 6856.474 |
| *BRAF* | 0.594 | 0.047 | 0.000 | 3662.089 |
| *BRCA1* | 0.648 | 0.047 | 0.000 | 22951.980 |
| *CCND2* | 0.542 | 0.044 | 0.000 | 990.442 |
| *CTCF* | 0.568 | 0.046 | 0.000 | 1763.214 |
| *CUL4B* | 0.127 | 5.146 | 0.626 | 42.279 |
| *EWSR1* | 0.577 | 0.046 | 0.000 | 2246.679 |
| *GLI1* | 0.594 | 0.047 | 0.000 | 3662.089 |
| *HIST1H1C* | 0.593 | 1.742 | 0.228 | 13.335 |
| *IRS2* | 0.617 | 0.047 | 0.000 | 7567.742 |
| *NEB* | 0.001 | 17.307 | 3.305 | 90.639 |
| *NOTCH1* | 0.577 | 0.046 | 0.000 | 2246.679 |
| *PARP3* | 0.617 | 0.047 | 0.000 | 7567.742 |
| *PIK3CG* | 0.446 | 2.212 | 0.287 | 17.079 |
| *PTPRT* | 0.648 | 0.047 | 0.000 | 22951.980 |
| *RNF43* | 0.594 | 0.047 | 0.000 | 3662.089 |
| *RUNX1* | 0.442 | 2.223 | 0.290 | 17.052 |
| *SMAD2* | 0.272 | 3.194 | 0.403 | 25.307 |
| *STAT5B* | 0.594 | 0.047 | 0.000 | 3662.089 |
| *SYK* | 0.604 | 0.047 | 0.000 | 4929.702 |
| *TET1* | 0.594 | 0.047 | 0.000 | 3662.089 |
| *TET2* | 0.604 | 0.047 | 0.000 | 4929.702 |
| *TMPRSS2* | 0.604 | 0.047 | 0.000 | 4929.702 |
| *WT1* | 0.644 | 1.615 | 0.212 | 12.328 |
| *ABL1* | 0.577 | 0.046 | 0.000 | 2246.679 |
| *ALOX12B* | 0.604 | 0.047 | 0.000 | 4929.702 |
| *BCL2L1* | 0.568 | 0.046 | 0.000 | 1763.214 |
| *BCL6* | 0.614 | 0.047 | 0.000 | 6856.474 |
| *BCOR* | 0.644 | 1.615 | 0.212 | 12.328 |
| *BRD4* | 0.604 | 0.047 | 0.000 | 4929.702 |
| *CASP8* | 0.483 | 2.069 | 0.271 | 15.781 |
| *CDKN1C* | 0.568 | 0.046 | 0.000 | 1763.214 |
| *CHD2* | 0.542 | 0.044 | 0.000 | 990.442 |
| *DNMT3B* | 0.648 | 0.047 | 0.000 | 22951.980 |
| *EPHA7* | 0.542 | 0.044 | 0.000 | 990.442 |
| *FH* | 0.577 | 0.046 | 0.000 | 2246.679 |
| *FLT1* | 0.568 | 0.046 | 0.000 | 1763.214 |
| *FUBP1* | 0.604 | 0.047 | 0.000 | 4929.702 |
| *GATA2* | 0.594 | 0.047 | 0.000 | 3662.089 |
| *GNAS* | 0.594 | 0.047 | 0.000 | 3662.089 |
| *HIST1H3I* | 0.331 | 2.739 | 0.359 | 20.924 |
| *HNF1B* | 0.331 | 2.739 | 0.359 | 20.924 |
| *INHBA* | 0.518 | 1.960 | 0.255 | 15.061 |
| *INPP4B* | 0.289 | 3.010 | 0.392 | 23.121 |
| *KDM5C* | 0.127 | 5.146 | 0.626 | 42.279 |
| *MAP3K1* | 0.604 | 0.047 | 0.000 | 4929.702 |
| *MED12* | 0.604 | 0.047 | 0.000 | 4929.702 |
| *MPL* | 0.289 | 3.010 | 0.392 | 23.121 |
| *MYC* | 0.604 | 0.047 | 0.000 | 4929.702 |
| *NFKBIA* | 0.442 | 2.223 | 0.290 | 17.052 |
| *PALB2* | 0.028 | 5.421 | 1.195 | 24.597 |
| *PIK3C2B* | 0.568 | 0.046 | 0.000 | 1763.214 |
| *PIK3C2G* | 0.594 | 0.047 | 0.000 | 3662.089 |
| *RANBP2* | 0.604 | 0.047 | 0.000 | 4929.702 |
| *RASA1* | 0.568 | 0.046 | 0.000 | 1763.214 |
| *SOX2* | 0.568 | 0.046 | 0.000 | 1763.214 |
| *TRAF2* | 0.604 | 0.047 | 0.000 | 4929.702 |
| *FGF3* | 0.722 | 0.048 | 0.000 | 878045.307 |
| *ERCC1* | 0.707 | 0.048 | 0.000 | 362706.060 |
| *SH2B3* | 0.669 | 0.047 | 0.000 | 56546.993 |
| *PGR* | 0.707 | 0.048 | 0.000 | 362706.060 |
| *EMSY* | 0.707 | 0.048 | 0.000 | 362706.060 |
| *FANCD2* | 0.796 | 0.048 | 0.000 | 472327077.897 |
| *FGFR1* | 0.029 | 10.924 | 1.276 | 93.521 |
| *IGF1R* | 0.669 | 0.047 | 0.000 | 56546.993 |
| *INSR* | 0.707 | 0.048 | 0.000 | 362706.060 |
| *KDM5A* | 0.722 | 0.048 | 0.000 | 878045.307 |
| *KRAS* | 0.707 | 0.048 | 0.000 | 362706.060 |
| *MET* | 0.707 | 0.048 | 0.000 | 362706.060 |
| *MLH1* | 0.011 | 18.580 | 1.933 | 178.630 |
| *MRE11* | 0.796 | 0.048 | 0.000 | 472327077.897 |
| *RAF1* | 0.042 | 8.986 | 1.082 | 74.659 |
| *RUNX1T1* | 0.669 | 0.047 | 0.000 | 56546.993 |
| *TNFAIP3* | 0.076 | 6.561 | 0.820 | 52.477 |
| *TNFRSF14* | 0.669 | 0.047 | 0.000 | 56546.993 |
| *ALK* | 0.796 | 0.048 | 0.000 | 472327077.897 |
| *ANKRD11* | 0.796 | 0.048 | 0.000 | 472327077.897 |
| *CARD11* | 0.796 | 0.048 | 0.000 | 472327077.897 |
| *CDKN1B* | 0.011 | 18.580 | 1.933 | 178.630 |
| *CRLF2* | 0.356 | 2.628 | 0.338 | 20.423 |
| *CUL3* | 0.707 | 0.048 | 0.000 | 362706.060 |
| *EPHB1* | 0.019 | 13.794 | 1.542 | 123.422 |
| *ERRFI1* | 0.707 | 0.048 | 0.000 | 362706.060 |
| *GALNT12* | 0.707 | 0.048 | 0.000 | 362706.060 |
| *GRIN2A* | 0.722 | 0.048 | 0.000 | 878045.307 |
| *HSP90AA1* | 0.722 | 0.048 | 0.000 | 878045.307 |
| *IKZF1* | 0.707 | 0.048 | 0.000 | 362706.060 |
| *KEAP1* | 0.707 | 0.048 | 0.000 | 362706.060 |
| *MST1* | 0.722 | 0.048 | 0.000 | 878045.307 |
| *NOTCH2* | 0.722 | 0.048 | 0.000 | 878045.307 |
| *NOTCH3* | 0.707 | 0.048 | 0.000 | 362706.060 |
| *NTRK1* | 0.669 | 0.047 | 0.000 | 56546.993 |
| *NTRK2* | 0.722 | 0.048 | 0.000 | 878045.307 |
| *PDGFRB* | 0.669 | 0.047 | 0.000 | 56546.993 |
| *PIK3R2* | 0.707 | 0.048 | 0.000 | 362706.060 |
| *PPP2R1A* | 0.825 | 0.048 | 0.000 | 23090443987.304 |
| *PPP6C* | 0.707 | 0.048 | 0.000 | 362706.060 |
| *PRKAR1A* | 0.669 | 0.047 | 0.000 | 56546.993 |
| *RAD51* | 0.707 | 0.048 | 0.000 | 362706.060 |
| *RAD52* | 0.722 | 0.048 | 0.000 | 878045.307 |
| *TIPARP* | 0.076 | 6.561 | 0.820 | 52.477 |
| *ZFHX3* | 0.707 | 0.048 | 0.000 | 362706.060 |
| *AMER1* | 0.707 | 0.048 | 0.000 | 362706.060 |
| *AR* | 0.722 | 0.048 | 0.000 | 878045.307 |
| *ARID5B* | 0.796 | 0.048 | 0.000 | 472327077.897 |
| *AURKB* | 0.669 | 0.047 | 0.000 | 56546.993 |
| *B2M* | 0.707 | 0.048 | 0.000 | 362706.060 |
| *BCL10* | 0.796 | 0.048 | 0.000 | 472327077.897 |
| *CCND3* | 0.707 | 0.048 | 0.000 | 362706.060 |
| *CCNE1* | 0.722 | 0.048 | 0.000 | 878045.307 |
| *CEBPA* | 0.669 | 0.047 | 0.000 | 56546.993 |
| *CHD1* | 0.707 | 0.048 | 0.000 | 362706.060 |
| *CHEK1* | 0.669 | 0.047 | 0.000 | 56546.993 |
| *CSF3R* | 0.172 | 4.146 | 0.539 | 31.903 |
| *CXCR4* | 0.707 | 0.048 | 0.000 | 362706.060 |
| *DIS3* | 0.707 | 0.048 | 0.000 | 362706.060 |
| *DNAJB1* | 0.707 | 0.048 | 0.000 | 362706.060 |
| *EPHA2* | 0.669 | 0.047 | 0.000 | 56546.993 |
| *ERCC4* | 0.722 | 0.048 | 0.000 | 878045.307 |
| *FANCE* | 0.707 | 0.048 | 0.000 | 362706.060 |
| *FANCF* | 0.707 | 0.048 | 0.000 | 362706.060 |
| *FGF12* | 0.011 | 18.580 | 1.933 | 178.630 |
| *FGFR4* | 0.707 | 0.048 | 0.000 | 362706.060 |
| *FOXP1* | 0.707 | 0.048 | 0.000 | 362706.060 |
| *GNA11* | 0.722 | 0.048 | 0.000 | 878045.307 |
| *GNAQ* | 0.707 | 0.048 | 0.000 | 362706.060 |
| *H3F3A* | 0.707 | 0.048 | 0.000 | 362706.060 |
| *H3F3C* | 0.076 | 6.561 | 0.820 | 52.477 |
| *HGF* | 0.707 | 0.048 | 0.000 | 362706.060 |
| *HIST1H3E* | 0.669 | 0.047 | 0.000 | 56546.993 |
| *HIST1H3J* | 0.172 | 4.146 | 0.539 | 31.903 |
| *IGF2* | 0.825 | 0.048 | 0.000 | 23090443987.304 |
| *IRS1* | 0.669 | 0.047 | 0.000 | 56546.993 |
| *KAT6A* | 0.707 | 0.048 | 0.000 | 362706.060 |
| *KIT* | 0.097 | 5.751 | 0.728 | 45.419 |
| *LATS2* | 0.097 | 5.751 | 0.728 | 45.419 |
| *MAGI2* | 0.722 | 0.048 | 0.000 | 878045.307 |
| *MAP3K13* | 0.669 | 0.047 | 0.000 | 56546.993 |
| *MAX* | 0.029 | 10.924 | 1.276 | 93.521 |
| *MDM4* | 0.669 | 0.047 | 0.000 | 56546.993 |
| *MEF2B* | 0.707 | 0.048 | 0.000 | 362706.060 |
| *MSH2* | 0.669 | 0.047 | 0.000 | 56546.993 |
| *MSH6* | 0.076 | 6.561 | 0.820 | 52.477 |
| *MUTYH* | 0.097 | 5.751 | 0.728 | 45.419 |
| *NCOA3* | 0.707 | 0.048 | 0.000 | 362706.060 |
| *NFE2L2* | 0.097 | 5.751 | 0.728 | 45.419 |
| *NKX2_1* | 0.011 | 18.580 | 1.933 | 178.630 |
| *NOTCH4* | 0.669 | 0.047 | 0.000 | 56546.993 |
| *NRAS* | 0.722 | 0.048 | 0.000 | 878045.307 |
| *NTRK3* | 0.796 | 0.048 | 0.000 | 472327077.897 |
| *PAK5* | 0.669 | 0.047 | 0.000 | 56546.993 |
| *PARP4* | 0.669 | 0.047 | 0.000 | 56546.993 |
| *PAX5* | 0.796 | 0.048 | 0.000 | 472327077.897 |
| *PIK3C3* | 0.019 | 13.794 | 1.542 | 123.422 |
| *PIK3CB* | 0.722 | 0.048 | 0.000 | 878045.307 |
| *POLD1* | 0.707 | 0.048 | 0.000 | 362706.060 |
| *POM121L12* | 0.707 | 0.048 | 0.000 | 362706.060 |
| *PTK2* | 0.707 | 0.048 | 0.000 | 362706.060 |
| *RECQL4* | 0.042 | 8.986 | 1.082 | 74.659 |
| *RET* | 0.011 | 18.580 | 1.933 | 178.630 |
| *RHOA* | 0.669 | 0.047 | 0.000 | 56546.993 |
| *RIT1* | 0.796 | 0.048 | 0.000 | 472327077.897 |
| *SDHAF2* | 0.042 | 8.986 | 1.082 | 74.659 |
| *SDHB* | 0.011 | 18.580 | 1.933 | 178.630 |
| *SLX4* | 0.669 | 0.047 | 0.000 | 56546.993 |
| *SMARCB1* | 0.707 | 0.048 | 0.000 | 362706.060 |
| *SMARCD1* | 0.722 | 0.048 | 0.000 | 878045.307 |
| *SOX10* | 0.707 | 0.048 | 0.000 | 362706.060 |
| *TACC3* | 0.722 | 0.048 | 0.000 | 878045.307 |
| *TAF1* | 0.011 | 18.580 | 1.933 | 178.630 |
| *TCF3* | 0.707 | 0.048 | 0.000 | 362706.060 |
| *TENT5C* | 0.669 | 0.047 | 0.000 | 56546.993 |
| *TGFBR2* | 0.722 | 0.048 | 0.000 | 878045.307 |
| *TNFSF11* | 0.076 | 6.561 | 0.820 | 52.477 |
| *TP63* | 0.707 | 0.048 | 0.000 | 362706.060 |
| *VEGFA* | 0.722 | 0.048 | 0.000 | 878045.307 |
| *WRN* | 0.707 | 0.048 | 0.000 | 362706.060 |
| *XIAP* | 0.796 | 0.048 | 0.000 | 472327077.897 |
| *XPO1* | 0.722 | 0.048 | 0.000 | 878045.307 |
| *ZNF703* | 0.019 | 13.794 | 1.542 | 123.422 |
| *ZNRF3* | 0.669 | 0.047 | 0.000 | 56546.993 |
| *CCND1* | NA | NA | NA | NA |
| *FGF19* | NA | NA | NA | NA |
| *FGF4* | NA | NA | NA | NA |
| *CDKN2A* | NA | NA | NA | NA |
| *CDKN2B* | NA | NA | NA | NA |
| *MDM2* | NA | NA | NA | NA |
| *HNF1A* | NA | NA | NA | NA |
| *TOP1* | NA | NA | NA | NA |
| *TSC2* | NA | NA | NA | NA |
| *ACVR1B* | NA | NA | NA | NA |
| *AXIN1* | NA | NA | NA | NA |
| *BACH1* | NA | NA | NA | NA |
| *CD79A* | NA | NA | NA | NA |
| *CD79B* | NA | NA | NA | NA |
| *CDK4* | NA | NA | NA | NA |
| *CDK6* | NA | NA | NA | NA |
| *CYLD* | NA | NA | NA | NA |
| *EPHA3* | NA | NA | NA | NA |
| *FANCL* | NA | NA | NA | NA |
| *FGFR2* | NA | NA | NA | NA |
| *GREM1* | NA | NA | NA | NA |
| *HLA_A* | NA | NA | NA | NA |
| *MAP2K2* | NA | NA | NA | NA |
| *NF2* | NA | NA | NA | NA |
| *NKX3_1* | NA | NA | NA | NA |
| *NR4A3* | NA | NA | NA | NA |
| *NTHL1* | NA | NA | NA | NA |
| *NUP93* | NA | NA | NA | NA |
| *PDGFRA* | NA | NA | NA | NA |
| *PIK3CD* | NA | NA | NA | NA |
| *PRDM1* | NA | NA | NA | NA |
| *PRKN* | NA | NA | NA | NA |
| *RAC1* | NA | NA | NA | NA |
| *RARA* | NA | NA | NA | NA |
| *SOX9* | NA | NA | NA | NA |
| *SRC* | NA | NA | NA | NA |
| *STAT3* | NA | NA | NA | NA |
| *STK40* | NA | NA | NA | NA |
| *SUFU* | NA | NA | NA | NA |
| *ZBTB2* | NA | NA | NA | NA |

CI: confidence interval; NA: not available.

**Table S5.** Univariate analysis of BCG treated NMIBC subgroup (n=46)

| Gene | P | Hazard ratio | Lower limit of 95% CI | Upper limit of 95% CI |
| --- | --- | --- | --- | --- |
| *KDM6A* | 0.241 | 0.436 | 0.109 | 1.745 |
| *KMT2D* | 0.994 | 0.995 | 0.267 | 3.708 |
| *FGFR3* | 0.737 | 0.788 | 0.197 | 3.153 |
| *KMT2C* | 0.120 | 2.841 | 0.762 | 10.585 |
| *STAG2* | 0.481 | 1.604 | 0.430 | 5.975 |
| *CREBBP* | 0.189 | 0.248 | 0.031 | 1.983 |
| *PIK3CA* | 0.290 | 2.035 | 0.545 | 7.596 |
| *TP53* | 0.730 | 1.276 | 0.319 | 5.104 |
| *ARID1A* | 0.291 | 0.326 | 0.041 | 2.610 |
| *TERT* | 0.071 | 3.376 | 0.901 | 12.641 |
| *ERBB2* | 0.801 | 0.765 | 0.096 | 6.120 |
| *FANCA* | 0.484 | 1.753 | 0.364 | 8.451 |
| *FAT3* | 0.355 | 0.035 | 0.000 | 41.969 |
| *ATM* | 0.833 | 0.799 | 0.100 | 6.393 |
| *BAP1* | 0.389 | 0.037 | 0.000 | 66.632 |
| *LRP1B* | 0.732 | 0.696 | 0.087 | 5.565 |
| *KMT2A* | 0.946 | 0.931 | 0.116 | 7.452 |
| *ATR* | 0.471 | 0.041 | 0.000 | 244.607 |
| *NSD1* | 0.415 | 1.923 | 0.399 | 9.273 |
| *RBM10* | 0.448 | 0.040 | 0.000 | 163.499 |
| *SF3B1* | 0.755 | 0.718 | 0.090 | 5.747 |
| *TSC1* | 0.705 | 0.669 | 0.084 | 5.351 |
| *FBXW7* | 0.859 | 1.208 | 0.151 | 9.684 |
| *FAT1* | 0.951 | 1.067 | 0.133 | 8.548 |
| *NBN* | 0.951 | 1.067 | 0.133 | 8.548 |
| *PRKDC* | 0.266 | 2.444 | 0.506 | 11.799 |
| *TRRAP* | 0.813 | 0.778 | 0.097 | 6.224 |
| *U2AF1* | 0.868 | 0.838 | 0.105 | 6.707 |
| *CTNNB1* | 0.718 | 1.466 | 0.183 | 11.733 |
| *PMS1* | 0.813 | 0.778 | 0.097 | 6.224 |
| *EP300* | 0.521 | 0.042 | 0.000 | 655.322 |
| *SMARCA4* | 0.936 | 0.919 | 0.115 | 7.352 |
| *ARID1B* | 0.978 | 1.030 | 0.129 | 8.235 |
| *BRCA2* | 0.494 | 0.042 | 0.000 | 377.771 |
| *ERBB3* | 0.471 | 0.041 | 0.000 | 244.607 |
| *NF1* | 0.980 | 0.973 | 0.122 | 7.785 |
| *APC* | 0.215 | 2.724 | 0.559 | 13.271 |
| *BRIP1* | 0.818 | 1.276 | 0.159 | 10.208 |
| *CDK12* | 0.871 | 1.188 | 0.149 | 9.511 |
| *FANCI* | 0.607 | 1.725 | 0.215 | 13.816 |
| *CHEK2* | 0.639 | 1.646 | 0.206 | 13.179 |
| *HRAS* | 0.548 | 0.043 | 0.000 | 1216.564 |
| *INPP4A* | 0.999 | 1.001 | 0.125 | 8.009 |
| *ASXL2* | 0.546 | 1.898 | 0.237 | 15.206 |
| *BLM* | 0.735 | 1.433 | 0.178 | 11.508 |
| *CIC* | 0.613 | 0.045 | 0.000 | 7415.321 |
| *JAK3* | 0.621 | 0.045 | 0.000 | 9671.601 |
| *PBRM1* | 0.521 | 0.042 | 0.000 | 655.322 |
| *RAD51C* | 0.548 | 0.043 | 0.000 | 1216.564 |
| *SETD2* | 0.548 | 0.043 | 0.000 | 1216.564 |
| *AKT1* | 0.793 | 1.322 | 0.165 | 10.577 |
| *ARID2* | 0.548 | 0.043 | 0.000 | 1216.564 |
| *ASXL1* | 0.793 | 1.322 | 0.165 | 10.577 |
| *CDKN1A* | 0.580 | 0.044 | 0.000 | 2766.444 |
| *CSF1R* | 0.546 | 1.898 | 0.237 | 15.206 |
| *MLH3* | 0.818 | 1.276 | 0.159 | 10.208 |
| *ZNF217* | 0.613 | 0.045 | 0.000 | 7415.321 |
| *TBX3* | 0.445 | 2.261 | 0.278 | 18.364 |
| *ERCC2* | 0.580 | 0.044 | 0.000 | 2766.444 |
| *SDHC* | 0.003 | 11.892 | 2.272 | 62.246 |
| *ERBB4* | 0.546 | 1.898 | 0.237 | 15.206 |
| *PTCH1* | 0.613 | 0.045 | 0.000 | 7415.321 |
| *DAXX* | 0.607 | 1.725 | 0.215 | 13.816 |
| *DOT1L* | 0.580 | 0.044 | 0.000 | 2766.444 |
| *EGFR* | 0.696 | 0.047 | 0.000 | 218142.954 |
| *JUN* | 0.653 | 0.046 | 0.000 | 30882.789 |
| *SMO* | 0.653 | 0.046 | 0.000 | 30882.789 |
| *ATRX* | 0.233 | 3.593 | 0.439 | 29.432 |
| *CHD4* | 0.653 | 0.046 | 0.000 | 30882.789 |
| *FLT4* | 0.670 | 1.572 | 0.196 | 12.576 |
| *JAK2* | 0.580 | 0.044 | 0.000 | 2766.444 |
| *KDR* | 0.580 | 0.044 | 0.000 | 2766.444 |
| *MST1R* | 0.696 | 0.047 | 0.000 | 218142.954 |
| *NCOR1* | 0.580 | 0.044 | 0.000 | 2766.444 |
| *PMS2* | 0.613 | 0.045 | 0.000 | 7415.321 |
| *PREX2* | 0.607 | 1.725 | 0.215 | 13.816 |
| *PTPRD* | 0.653 | 0.046 | 0.000 | 30882.789 |
| *RAD51D* | 0.752 | 0.048 | 0.000 | 7169472.676 |
| *RPTOR* | 0.580 | 0.044 | 0.000 | 2766.444 |
| *ABL2* | 0.546 | 1.898 | 0.237 | 15.206 |
| *BCORL1* | 0.580 | 0.044 | 0.000 | 2766.444 |
| *EPHA5* | 0.580 | 0.044 | 0.000 | 2766.444 |
| *FLT3* | 0.488 | 2.088 | 0.261 | 16.730 |
| *FOXA1* | 0.752 | 0.048 | 0.000 | 7169472.676 |
| *GATA3* | 0.168 | 4.540 | 0.527 | 39.084 |
| *GNA13* | 0.653 | 0.046 | 0.000 | 30882.789 |
| *IDH1* | 0.580 | 0.044 | 0.000 | 2766.444 |
| *JAK1* | 0.580 | 0.044 | 0.000 | 2766.444 |
| *LATS1* | 0.580 | 0.044 | 0.000 | 2766.444 |
| *MCL1* | 0.369 | 2.594 | 0.324 | 20.806 |
| *MSH3* | 0.653 | 0.046 | 0.000 | 30882.789 |
| *MTOR* | 0.024 | 6.301 | 1.278 | 31.072 |
| *POLE* | 0.752 | 0.048 | 0.000 | 7169472.676 |
| *PTEN* | 0.653 | 0.046 | 0.000 | 30882.789 |
| *PTPRS* | 0.546 | 1.898 | 0.237 | 15.206 |
| *RAD50* | 0.460 | 2.191 | 0.274 | 17.556 |
| *RAD54L* | 0.613 | 0.045 | 0.000 | 7415.321 |
| *RB1* | 0.517 | 1.991 | 0.249 | 15.951 |
| *RICTOR* | 0.580 | 0.044 | 0.000 | 2766.444 |
| *SPEN* | 0.653 | 0.046 | 0.000 | 30882.789 |
| *SPTA1* | 0.209 | 3.809 | 0.472 | 30.713 |
| *DDR2* | 0.752 | 0.048 | 0.000 | 7169472.676 |
| *PLCG2* | 0.653 | 0.046 | 0.000 | 30882.789 |
| *PTPN11* | 0.168 | 4.540 | 0.527 | 39.084 |
| *PAK1* | 0.335 | 2.788 | 0.347 | 22.400 |
| *ROS1* | 0.131 | 4.967 | 0.621 | 39.740 |
| *ADGRA2* | 0.653 | 0.046 | 0.000 | 30882.789 |
| *BRAF* | 0.653 | 0.046 | 0.000 | 30882.789 |
| *BRCA1* | 0.696 | 0.047 | 0.000 | 218142.954 |
| *CCND2* | 0.653 | 0.046 | 0.000 | 30882.789 |
| *CTCF* | 0.653 | 0.046 | 0.000 | 30882.789 |
| *CUL4B* | 0.090 | 6.752 | 0.743 | 61.374 |
| *EWSR1* | 0.653 | 0.046 | 0.000 | 30882.789 |
| *GLI1* | 0.653 | 0.046 | 0.000 | 30882.789 |
| *HIST1H1C* | 0.335 | 2.788 | 0.347 | 22.400 |
| *IRS2* | 0.820 | 0.048 | 0.000 | 11047880895.330 |
| *NEB* | 0.001 | 21.213 | 3.466 | 129.823 |
| *NOTCH1* | 0.653 | 0.046 | 0.000 | 30882.789 |
| *PARP3* | 0.696 | 0.047 | 0.000 | 218142.954 |
| *PIK3CG* | 0.752 | 0.048 | 0.000 | 7169472.676 |
| *PTPRT* | 0.696 | 0.047 | 0.000 | 218142.954 |
| *RNF43* | 0.752 | 0.048 | 0.000 | 7169472.676 |
| *RUNX1* | 0.335 | 2.788 | 0.347 | 22.400 |
| *SMAD2* | 0.200 | 4.014 | 0.480 | 33.597 |
| *STAT5B* | 0.752 | 0.048 | 0.000 | 7169472.676 |
| *SYK* | 0.653 | 0.046 | 0.000 | 30882.789 |
| *TET1* | 0.653 | 0.046 | 0.000 | 30882.789 |
| *TET2* | 0.653 | 0.046 | 0.000 | 30882.789 |
| *TMPRSS2* | 0.653 | 0.046 | 0.000 | 30882.789 |
| *WT1* | 0.369 | 2.594 | 0.324 | 20.806 |
| *ABL1* | 0.653 | 0.046 | 0.000 | 30882.789 |
| *ALOX12B* | 0.653 | 0.046 | 0.000 | 30882.789 |
| *BCL2L1* | 0.653 | 0.046 | 0.000 | 30882.789 |
| *BCL6* | 0.653 | 0.046 | 0.000 | 30882.789 |
| *BCOR* | 0.369 | 2.594 | 0.324 | 20.806 |
| *BRD4* | 0.653 | 0.046 | 0.000 | 30882.789 |
| *CASP8* | 0.335 | 2.788 | 0.347 | 22.400 |
| *CDKN1C* | 0.653 | 0.046 | 0.000 | 30882.789 |
| *CHD2* | 0.653 | 0.046 | 0.000 | 30882.789 |
| *DNMT3B* | 0.696 | 0.047 | 0.000 | 218142.954 |
| *EPHA7* | 0.653 | 0.046 | 0.000 | 30882.789 |
| *FH* | 0.653 | 0.046 | 0.000 | 30882.789 |
| *FLT1* | 0.653 | 0.046 | 0.000 | 30882.789 |
| *FUBP1* | 0.653 | 0.046 | 0.000 | 30882.789 |
| *GATA2* | 0.653 | 0.046 | 0.000 | 30882.789 |
| *GNAS* | 0.653 | 0.046 | 0.000 | 30882.789 |
| *HIST1H3I* | 0.752 | 0.048 | 0.000 | 7169472.676 |
| *HNF1B* | 0.752 | 0.048 | 0.000 | 7169472.676 |
| *INHBA* | 0.269 | 3.246 | 0.403 | 26.148 |
| *INPP4B* | 0.209 | 3.809 | 0.472 | 30.713 |
| *KDM5C* | 0.820 | 0.048 | 0.000 | 11047880895.330 |
| *MAP3K1* | 0.653 | 0.046 | 0.000 | 30882.789 |
| *MED12* | 0.653 | 0.046 | 0.000 | 30882.789 |
| *MPL* | 0.209 | 3.809 | 0.472 | 30.713 |
| *MYC* | 0.653 | 0.046 | 0.000 | 30882.789 |
| *NFKBIA* | 0.335 | 2.788 | 0.347 | 22.400 |
| *PALB2* | 0.052 | 8.377 | 0.978 | 71.738 |
| *PIK3C2B* | 0.653 | 0.046 | 0.000 | 30882.789 |
| *PIK3C2G* | 0.752 | 0.048 | 0.000 | 7169472.676 |
| *RANBP2* | 0.653 | 0.046 | 0.000 | 30882.789 |
| *RASA1* | 0.653 | 0.046 | 0.000 | 30882.789 |
| *SOX2* | 0.653 | 0.046 | 0.000 | 30882.789 |
| *TRAF2* | 0.653 | 0.046 | 0.000 | 30882.789 |
| *FGF3* | 0.752 | 0.048 | 0.000 | 7169472.676 |
| *ERCC1* | NA | NA | NA | NA |
| *SH2B3* | 0.752 | 0.048 | 0.000 | 7169472.676 |
| *PGR* | 0.752 | 0.048 | 0.000 | 7169472.676 |
| *EMSY* | 0.752 | 0.048 | 0.000 | 7169472.676 |
| *FANCD2* | 0.820 | 0.048 | 0.000 | 11047880895.330 |
| *FGFR1* | 0.021 | 14.493 | 1.507 | 139.347 |
| *IGF1R* | NA | NA | NA | NA |
| *INSR* | 0.752 | 0.048 | 0.000 | 7169472.676 |
| *KDM5A* | 0.752 | 0.048 | 0.000 | 7169472.676 |
| *KRAS* | 0.752 | 0.048 | 0.000 | 7169472.676 |
| *MET* | 0.752 | 0.048 | 0.000 | 7169472.676 |
| *MLH1* | NA | NA | NA | NA |
| *MRE11* | 0.820 | 0.048 | 0.000 | 11047880895.330 |
| *RAF1* | 0.034 | 10.686 | 1.194 | 95.634 |
| *RUNX1T1* | 0.752 | 0.048 | 0.000 | 7169472.676 |
| *TNFAIP3* | 0.052 | 8.377 | 0.978 | 71.738 |
| *TNFRSF14* | 0.752 | 0.048 | 0.000 | 7169472.676 |
| *ALK* | 0.820 | 0.048 | 0.000 | 11047880895.330 |
| *ANKRD11* | 0.820 | 0.048 | 0.000 | 11047880895.330 |
| *CARD11* | 0.820 | 0.048 | 0.000 | 11047880895.330 |
| *CDKN1B* | NA | NA | NA | NA |
| *CRLF2* | NA | NA | NA | NA |
| *CUL3* | 0.752 | 0.048 | 0.000 | 7169472.676 |
| *EPHB1* | NA | NA | NA | NA |
| *ERRFI1* | 0.752 | 0.048 | 0.000 | 7169472.676 |
| *GALNT12* | 0.752 | 0.048 | 0.000 | 7169472.676 |
| *GRIN2A* | 0.752 | 0.048 | 0.000 | 7169472.676 |
| *HSP90AA1* | 0.752 | 0.048 | 0.000 | 7169472.676 |
| *IKZF1* | 0.752 | 0.048 | 0.000 | 7169472.676 |
| *KEAP1* | 0.752 | 0.048 | 0.000 | 7169472.676 |
| *MST1* | 0.752 | 0.048 | 0.000 | 7169472.676 |
| *NOTCH2* | 0.752 | 0.048 | 0.000 | 7169472.676 |
| *NOTCH3* | NA | NA | NA | NA |
| *NTRK1* | 0.752 | 0.048 | 0.000 | 7169472.676 |
| *NTRK2* | 0.752 | 0.048 | 0.000 | 7169472.676 |
| *PDGFRB* | 0.752 | 0.048 | 0.000 | 7169472.676 |
| *PIK3R2* | 0.752 | 0.048 | 0.000 | 7169472.676 |
| *PPP2R1A* | 0.840 | 0.048 | 0.000 | 309088017370.675 |
| *PPP6C* | 0.752 | 0.048 | 0.000 | 7169472.676 |
| *PRKAR1A* | 0.752 | 0.048 | 0.000 | 7169472.676 |
| *RAD51* | 0.752 | 0.048 | 0.000 | 7169472.676 |
| *RAD52* | 0.752 | 0.048 | 0.000 | 7169472.676 |
| *TIPARP* | 0.052 | 8.377 | 0.978 | 71.738 |
| *ZFHX3* | 0.752 | 0.048 | 0.000 | 7169472.676 |
| *AMER1* | NA | NA | NA | NA |
| *AR* | 0.752 | 0.048 | 0.000 | 7169472.676 |
| *ARID5B* | 0.820 | 0.048 | 0.000 | 11047880895.330 |
| *AURKB* | 0.752 | 0.048 | 0.000 | 7169472.676 |
| *B2M* | 0.752 | 0.048 | 0.000 | 7169472.676 |
| *BCL10* | 0.820 | 0.048 | 0.000 | 11047880895.330 |
| *CCND3* | NA | NA | NA | NA |
| *CCNE1* | 0.752 | 0.048 | 0.000 | 7169472.676 |
| *CEBPA* | 0.752 | 0.048 | 0.000 | 7169472.676 |
| *CHD1* | 0.752 | 0.048 | 0.000 | 7169472.676 |
| *CHEK1* | 0.752 | 0.048 | 0.000 | 7169472.676 |
| *CSF3R* | NA | NA | NA | NA |
| *CXCR4* | 0.752 | 0.048 | 0.000 | 7169472.676 |
| *DIS3* | 0.752 | 0.048 | 0.000 | 7169472.676 |
| *DNAJB1* | 0.752 | 0.048 | 0.000 | 7169472.676 |
| *EPHA2* | NA | NA | NA | NA |
| *ERCC4* | 0.752 | 0.048 | 0.000 | 7169472.676 |
| *FANCE* | 0.752 | 0.048 | 0.000 | 7169472.676 |
| *FANCF* | 0.752 | 0.048 | 0.000 | 7169472.676 |
| *FGF12* | 0.012 | 21.995 | 1.994 | 242.587 |
| *FGFR4* | 0.752 | 0.048 | 0.000 | 7169472.676 |
| *FOXP1* | 0.752 | 0.048 | 0.000 | 7169472.676 |
| *GNA11* | 0.752 | 0.048 | 0.000 | 7169472.676 |
| *GNAQ* | 0.752 | 0.048 | 0.000 | 7169472.676 |
| *H3F3A* | 0.752 | 0.048 | 0.000 | 7169472.676 |
| *H3F3C* | 0.052 | 8.377 | 0.978 | 71.738 |
| *HGF* | 0.752 | 0.048 | 0.000 | 7169472.676 |
| *HIST1H3E* | 0.752 | 0.048 | 0.000 | 7169472.676 |
| *HIST1H3J* | NA | NA | NA | NA |
| *IGF2* | 0.840 | 0.048 | 0.000 | 309088017370.675 |
| *IRS1* | NA | NA | NA | NA |
| *KAT6A* | 0.752 | 0.048 | 0.000 | 7169472.676 |
| *KIT* | 0.075 | 6.853 | 0.825 | 56.959 |
| *LATS2* | 0.075 | 6.853 | 0.825 | 56.959 |
| *MAGI2* | 0.752 | 0.048 | 0.000 | 7169472.676 |
| *MAP3K13* | 0.752 | 0.048 | 0.000 | 7169472.676 |
| *MAX* | 0.021 | 14.493 | 1.507 | 139.347 |
| *MDM4* | 0.752 | 0.048 | 0.000 | 7169472.676 |
| *MEF2B* | 0.752 | 0.048 | 0.000 | 7169472.676 |
| *MSH2* | 0.752 | 0.048 | 0.000 | 7169472.676 |
| *MSH6* | 0.052 | 8.377 | 0.978 | 71.738 |
| *MUTYH* | 0.075 | 6.853 | 0.825 | 56.959 |
| *NCOA3* | 0.752 | 0.048 | 0.000 | 7169472.676 |
| *NFE2L2* | 0.075 | 6.853 | 0.825 | 56.959 |
| *NKX2_1* | NA | NA | NA | NA |
| *NOTCH4* | 0.752 | 0.048 | 0.000 | 7169472.676 |
| *NRAS* | 0.752 | 0.048 | 0.000 | 7169472.676 |
| *NTRK3* | 0.820 | 0.048 | 0.000 | 11047880895.330 |
| *PAK5* | 0.752 | 0.048 | 0.000 | 7169472.676 |
| *PARP4* | 0.752 | 0.048 | 0.000 | 7169472.676 |
| *PAX5* | 0.820 | 0.048 | 0.000 | 11047880895.330 |
| *PIK3C3* | NA | NA | NA | NA |
| *PIK3CB* | 0.752 | 0.048 | 0.000 | 7169472.676 |
| *POLD1* | 0.752 | 0.048 | 0.000 | 7169472.676 |
| *POM121L12* | 0.752 | 0.048 | 0.000 | 7169472.676 |
| *PTK2* | 0.752 | 0.048 | 0.000 | 7169472.676 |
| *RECQL4* | 0.034 | 10.686 | 1.194 | 95.634 |
| *RET* | NA | NA | NA | NA |
| *RHOA* | 0.752 | 0.048 | 0.000 | 7169472.676 |
| *RIT1* | 0.820 | 0.048 | 0.000 | 11047880895.330 |
| *SDHAF2* | 0.034 | 10.686 | 1.194 | 95.634 |
| *SDHB* | NA | NA | NA | NA |
| *SLX4* | 0.752 | 0.048 | 0.000 | 7169472.676 |
| *SMARCB1* | 0.752 | 0.048 | 0.000 | 7169472.676 |
| *SMARCD1* | 0.752 | 0.048 | 0.000 | 7169472.676 |
| *SOX10* | 0.752 | 0.048 | 0.000 | 7169472.676 |
| *TACC3* | 0.752 | 0.048 | 0.000 | 7169472.676 |
| *TAF1* | 0.012 | 21.995 | 1.994 | 242.587 |
| *TCF3* | 0.752 | 0.048 | 0.000 | 7169472.676 |
| *TENT5C* | 0.752 | 0.048 | 0.000 | 7169472.676 |
| *TGFBR2* | 0.752 | 0.048 | 0.000 | 7169472.676 |
| *TNFSF11* | 0.052 | 8.377 | 0.978 | 71.738 |
| *TP63* | 0.752 | 0.048 | 0.000 | 7169472.676 |
| *VEGFA* | 0.752 | 0.048 | 0.000 | 7169472.676 |
| *WRN* | 0.752 | 0.048 | 0.000 | 7169472.676 |
| *XIAP* | 0.820 | 0.048 | 0.000 | 11047880895.330 |
| *XPO1* | 0.752 | 0.048 | 0.000 | 7169472.676 |
| *ZNF703* | NA | NA | NA | NA |
| *ZNRF3* | 0.752 | 0.048 | 0.000 | 7169472.676 |
| *CCND1* | NA | NA | NA | NA |
| *FGF19* | NA | NA | NA | NA |
| *FGF4* | NA | NA | NA | NA |
| *CDKN2A* | NA | NA | NA | NA |
| *CDKN2B* | NA | NA | NA | NA |
| *MDM2* | NA | NA | NA | NA |
| *HNF1A* | NA | NA | NA | NA |
| *TOP1* | NA | NA | NA | NA |
| *TSC2* | NA | NA | NA | NA |
| *ACVR1B* | NA | NA | NA | NA |
| *AXIN1* | NA | NA | NA | NA |
| *BACH1* | NA | NA | NA | NA |
| *CD79A* | NA | NA | NA | NA |
| *CD79B* | NA | NA | NA | NA |
| *CDK4* | NA | NA | NA | NA |
| *CDK6* | NA | NA | NA | NA |
| *CYLD* | NA | NA | NA | NA |
| *EPHA3* | NA | NA | NA | NA |
| *FANCL* | NA | NA | NA | NA |
| *FGFR2* | NA | NA | NA | NA |
| *GREM1* | NA | NA | NA | NA |
| *HLA_A* | NA | NA | NA | NA |
| *MAP2K2* | NA | NA | NA | NA |
| *NF2* | NA | NA | NA | NA |
| *NKX3_1* | NA | NA | NA | NA |
| *NR4A3* | NA | NA | NA | NA |
| *NTHL1* | NA | NA | NA | NA |
| *NUP93* | NA | NA | NA | NA |
| *PDGFRA* | NA | NA | NA | NA |
| *PIK3CD* | NA | NA | NA | NA |
| *PRDM1* | NA | NA | NA | NA |
| *PRKN* | NA | NA | NA | NA |
| *RAC1* | NA | NA | NA | NA |
| *RARA* | NA | NA | NA | NA |
| *SOX9* | NA | NA | NA | NA |
| *SRC* | NA | NA | NA | NA |
| *STAT3* | NA | NA | NA | NA |
| *STK40* | NA | NA | NA | NA |
| *SUFU* | NA | NA | NA | NA |
| *ZBTB2* | NA | NA | NA | NA |

CI: confidence interval; NA: not available.

**Table A6.** Multivariate analysis of genetic mutations and recurrence survival rate

| Group | Gene | P value | Hazard ratio (95% CI) |
| --- | --- | --- | --- |
| Total patients (n=58) | ***NEB*** | 0.001 | 2082.619 (34.775 – 124725.435) |
|  | ***MLH1*** | 0.001 | 694.095 (16.048 – 30020.114) |
|  | ***FGF12*** | 0.001 | 694.095 (16.048 – 30020.114) |
|  | ***EPHB1*** | 0.023 | 127.565 (1.929 – 8437.869) |
|  | ***KDM6A*** | 0.794 | 0.000 (0.000 - 1.99892128170068E+25) |
|  | ***PALB2*** | 0.093 | 5.626 (0.752 - 42.087) |
|  | ***GATA3*** | 0.777 | 16772.652 (0.000 - 2.73680518564157E+33) |
|  | ***FGFR1*** | 0.007 | 84.087 (3.332 – 2122.167) |
|  | ***MTOR*** | 0.959 | 0.000 (0.000 – 2.06353024984906E+146) |
|  | ***RAF1*** | 0.977 | 173.515 (0.000 – 3.06240976585618E+152) |
| BCG subgroup (n=46) | ***NEB*** | 0.001 | 459.095 (10.880 – 19440.312) |
|  | ***FGF12*** | 0.960 | 64993.201 (0.000 - 5.44407404198896E+190) |
|  | ***FGFR1*** | 0.004 | 114.851 (4.720 – 2794.943) |
|  | ***SDHC*** | 0.017 | 18.583 (1.685 – 204.987) |
|  | ***MTOR*** | 0.969 | 0.000 (0.000 - 1.56770553356729E+182) |
|  | ***RAF1*** | 0.976 | 652.960 (0.000 - 5.46467759117583E+188) |

Mutated genes significantly correlated with prognosis were included into the multivariate analysis. Variants with 0 degrees of freedom in the analysis were not shown in the table. CI: confidence interval; NA: not available.

**Table S7.** Correlation between pathways and DDR genes mutation number and predicted models

| Genetic groups | P value | | |
| --- | --- | --- | --- |
|  | **Risk group stratification** | **CUETO score** | **EORTC score** |
| Epigenetic pathway | 0.697 | 0.591 | 0.024 |
| TP53/ Cell cycle pathway | 0.113 | 0.269 | 0.828 |
| RTK - PI3K pathway | 0.013 | 0.624 | 0.577 |
| Histone modification pathway | 0.697 | 0.317 | 0.039 |
| SWI/SNF pathway | 0.542 | 0.169 | 0.037 |
| DNA damage pathway | 0.404 | 0.394 | 0.087 |
| Alternative splicing pathway | 0.592 | 0.988 | 0.082 |
| DDR genes | 0.406 | 0.704 | 0.060 |

RTK: receptor tyrosine kinases; PI3K: phosphatidylinositol 3-kinases; SWI/SNF: Switch/ sucrose nonfermentable; DDR genes: DNA damage response and repair genes.

**Table S8.** DDR genes in Oncoscreen Plus Panel

| *ATM* | *ATR* | *ATRX* | *BARD1* | *BLM* | *BRCA1* | *BRCA2* | *BRIP1* | *CHEK1* | *CHEK2* | *CUL3* |
| --- | --- | --- | --- | --- | --- | --- | --- | --- | --- | --- |
| *CUL4A* | *ERCC1* | *ERCC2* | *ERCC3* | *FANCI* | *FANCL* | *FANCM* | *ERCC4* | *ERCC5* | *FAM175A* | *FANCA* |
| *FANCC* | *FANCD2* | *FANCE* | *FANCF* | *FANCG* | *IDH1* | *MDC1* | *MLH1* | *MLH3* | *MRE11A* | *MSH2* |
| *MSH3* | *MSH6* | *MUTYH* | *NBN* | *NTHL1* | *PALB2* | *PARP1* | *PARP2* | *PARP3* | *PARP4* | *PMS1* |
| *PMS2* | *POLD1* | *POLE* | *PRKDC* | *PTEN* | *RAD50* | *RAD51* | *RAD51B* | *RAD51C* | *RAD51D* | *RAD52* |
| *RAD54L* | *RECQL4* | *RPA1* | *SLX4* | *SMARCA4* | *TP53* | *WRN* | *XRCC2* | *XRCC3* |  |  |

**SUPPLEMENTARY FIGURE LEGENDS**

**Figure S1: Overview of oncoprint of 58 post-NMIBC patients part 1.** CN: copy number; EORTC: European Organization for Research and Treatment of Cancer; CUETO: Spanish Urological Club for Oncological Treatment; PS: prognostic model scoring; MN: mutation number.

**Figure S2: Overview of oncoprint of 58 post-NMIBC patients part 2.** CN: copy number; EORTC: European Organization for Research and Treatment of Cancer; CUETO: Spanish Urological Club for Oncological Treatment; PS: prognostic model scoring; MN: mutation number.

**Figure S3: Overview of oncoprint of 58 post-NMIBC patients part 3.** CN: copy number; EORTC: European Organization for Research and Treatment of Cancer; CUETO: Spanish Urological Club for Oncological Treatment; PS: prognostic model scoring; MN: mutation number.

**Figure S4: Overview of oncoprint of 58 post-NMIBC patients part 4.** CN: copy number; EORTC: European Organization for Research and Treatment of Cancer; CUETO: Spanish Urological Club for Oncological Treatment; PS: prognostic model scoring; MN: mutation number.

**Figure S5: Overview of oncoprint of 58 post-NMIBC patients part 5.** CN: copy number; EORTC: European Organization for Research and Treatment of Cancer; CUETO: Spanish Urological Club for Oncological Treatment; PS: prognostic model scoring; MN: mutation number.

**Figure S6: Overview of oncoprint of 58 post-NMIBC patients part 6.** CN: copy number; EORTC: European Organization for Research and Treatment of Cancer; CUETO: Spanish Urological Club for Oncological Treatment; PS: prognostic model scoring; MN: mutation number.

**Figure S7: Overview of oncoprint of 58 post-NMIBC patients part 7.** CN: copy number; EORTC: European Organization for Research and Treatment of Cancer; CUETO: Spanish Urological Club for Oncological Treatment; PS: prognostic model scoring; MN: mutation number.

**Figure S8: Oncoprint of DNA damage response and repair genes.** CN: copy number; DDR genes: DNA damage response and repair genes; TMB: tumor mutational burden; MND: mutation number of DDR genes.
