# Supplementary material for: Prognostic factors of non-muscle invasive bladder cancer: a study based on next-generation sequencing
Source: Cancer Cell Int. 2021 Jan 6;21:23. doi: 10.1186/s12935-020-01731-9 (PMC7789352; doi:10.1186/s12935-020-01731-9)
Supplement: Supplementary file 2 — Additional file 2: Figures S1 to S7. [file 12935_2020_1731_MOESM2_ESM.pdf]

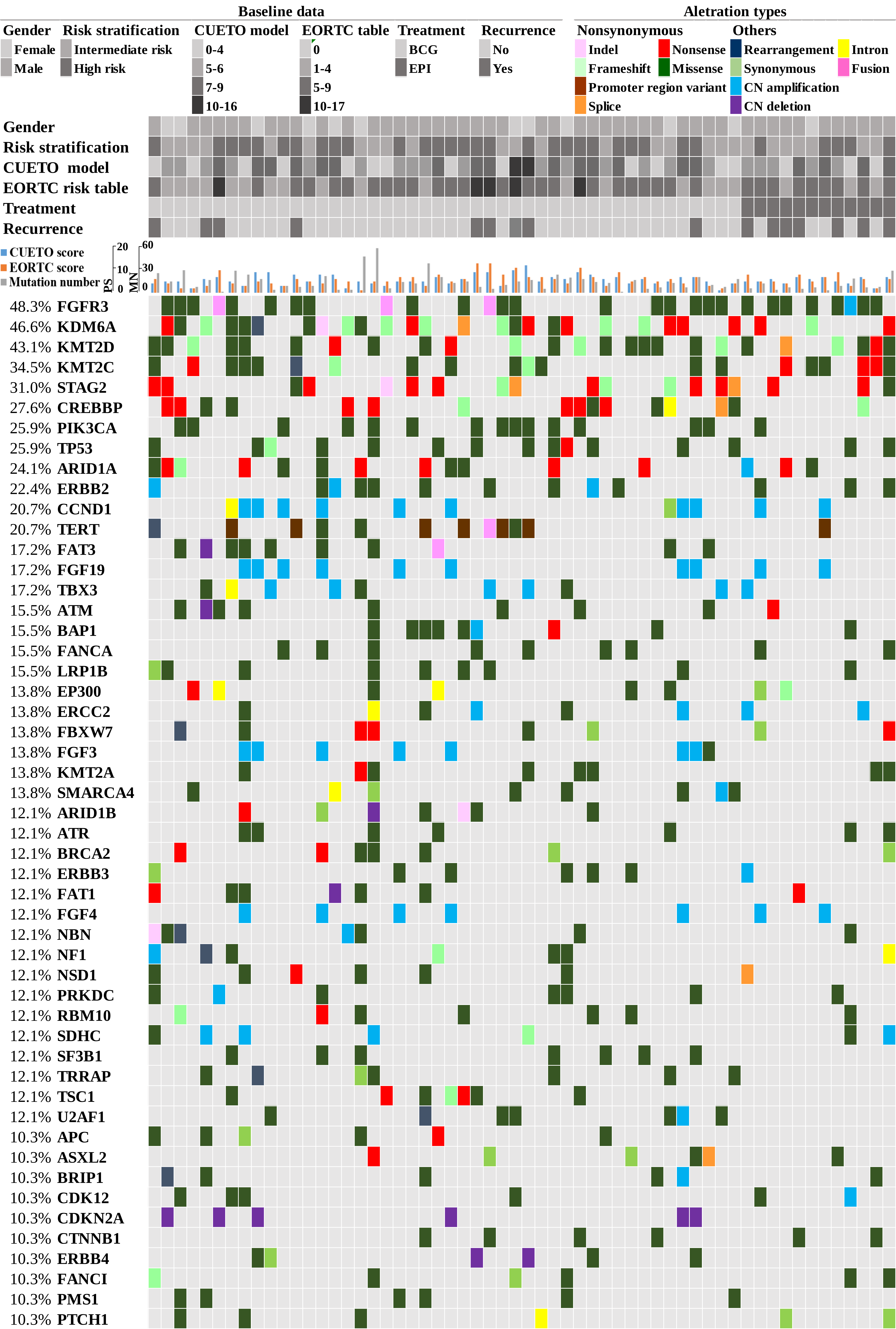

Baseline data
Aletration types
Gender
Female
Male
Risk stratification
Intermediate risk
High risk
CUETO model
0-4
5-6
7-9
10-16
EORTC table
0
1-4
5-9
10-17
Treatment
BCG
EPI
Recurrence
No
Yes
Nonsynonymous
Indel
Nonsense
Frameshift
Missense
Promoter region variant
Splice
Others
Rearrangement
Intron
Synonymous
Fusion
CN amplification
CN deletion
Gender
Risk stratification
CUETO model
EORTC risk table
Treatment
Recurrence
CUETO score
EORTC score
Mutation number
PS
MN
20
10
0
60
30
0
48.3% FGFR3
46.6% KDM6A
43.1% KMT2D
34.5% KMT2C
31.0% STAG2
27.6% CREBBP
25.9% PIK3CA
25.9% TP53
24.1% ARID1A
22.4% ERBB2
20.7% CCND1
20.7% TERT
17.2% FAT3
17.2% FGF19
17.2% TBX3
15.5% ATM
15.5% BAP1
15.5% FANCA
15.5% LRP1B
13.8% EP300
13.8% ERCC2
13.8% FBXW7
13.8% FGF3
13.8% KMT2A
13.8% SMARCA4
12.1% ARID1B
12.1% ATR
12.1% BRCA2
12.1% ERBB3
12.1% FAT1
12.1% FGF4
12.1% NBN
12.1% NF1
12.1% NSD1
12.1% PRKDC
12.1% RBM10
12.1% SDHC
12.1% SF3B1
12.1% TRRAP
12.1% TSC1
12.1% U2AF1
10.3% APC
10.3% ASXL2
10.3% BRIP1
10.3% CDK12
10.3% CDKN2A
10.3% CTNNB1
10.3% ERBB4
10.3% FANCI
10.3% PMS1
10.3% PTCH1

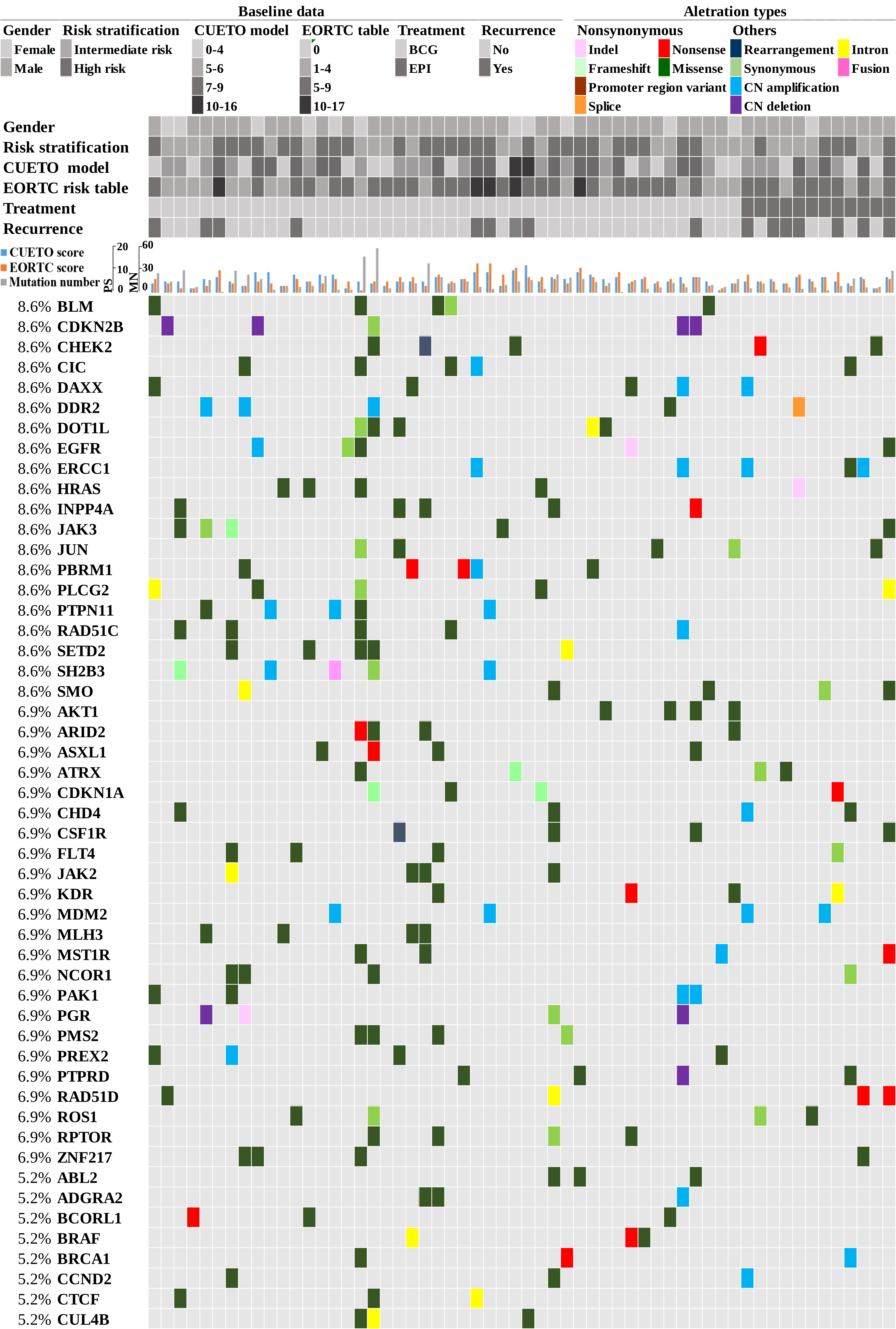
Baseline data
Aletration types
Gender
Female
Male
Risk stratification
Intermediate risk
High risk
CUETO model
0-4
5-6
7-9
10-16
EORTC table
0
1-4
5-9
10-17
Treatment
BCG
EPI
Recurrence
No
Yes
Nonsynonymous
Indel
Frameshift
Promoter region variant
Splice
Nonsense
Missense
Others
Rearrangement
Synonymous
CN amplification
CN deletion
Intron
Fusion
Gender
Risk stratification
CUETO model
EORTC risk table
Treatment
Recurrence
CUETO score
EORTC score
Mutation number
PS
MN
20
10
0
60
30
0
8.6% BLM
8.6% CDKN2B
8.6% CHEK2
8.6% CIC
8.6% DAXX
8.6% DDR2
8.6% DOT1L
8.6% EGFR
8.6% ERCC1
8.6% HRAS
8.6% INPP4A
8.6% JAK3
8.6% JUN
8.6% PBRM1
8.6% PLCG2
8.6% PTPN11
8.6% RAD51C
8.6% SETD2
8.6% SH2B3
8.6% SMO
6.9% AKT1
6.9% ARID2
6.9% ASXL1
6.9% ATRX
6.9% CDKN1A
6.9% CHD4
6.9% CSF1R
6.9% FLT4
6.9% JAK2
6.9% KDR
6.9% MDM2
6.9% MLH3
6.9% MST1R
6.9% NCOR1
6.9% PAK1
6.9% PGR
6.9% PMS2
6.9% PREX2
6.9% PTPRD
6.9% RAD51D
6.9% ROS1
6.9% RPTOR
6.9% ZNF217
5.2% ABL2
5.2% ADGRA2
5.2% BCORL1
5.2% BRAF
5.2% BRCA1
5.2% CCND2
5.2% CTCF
5.2% CUL4B

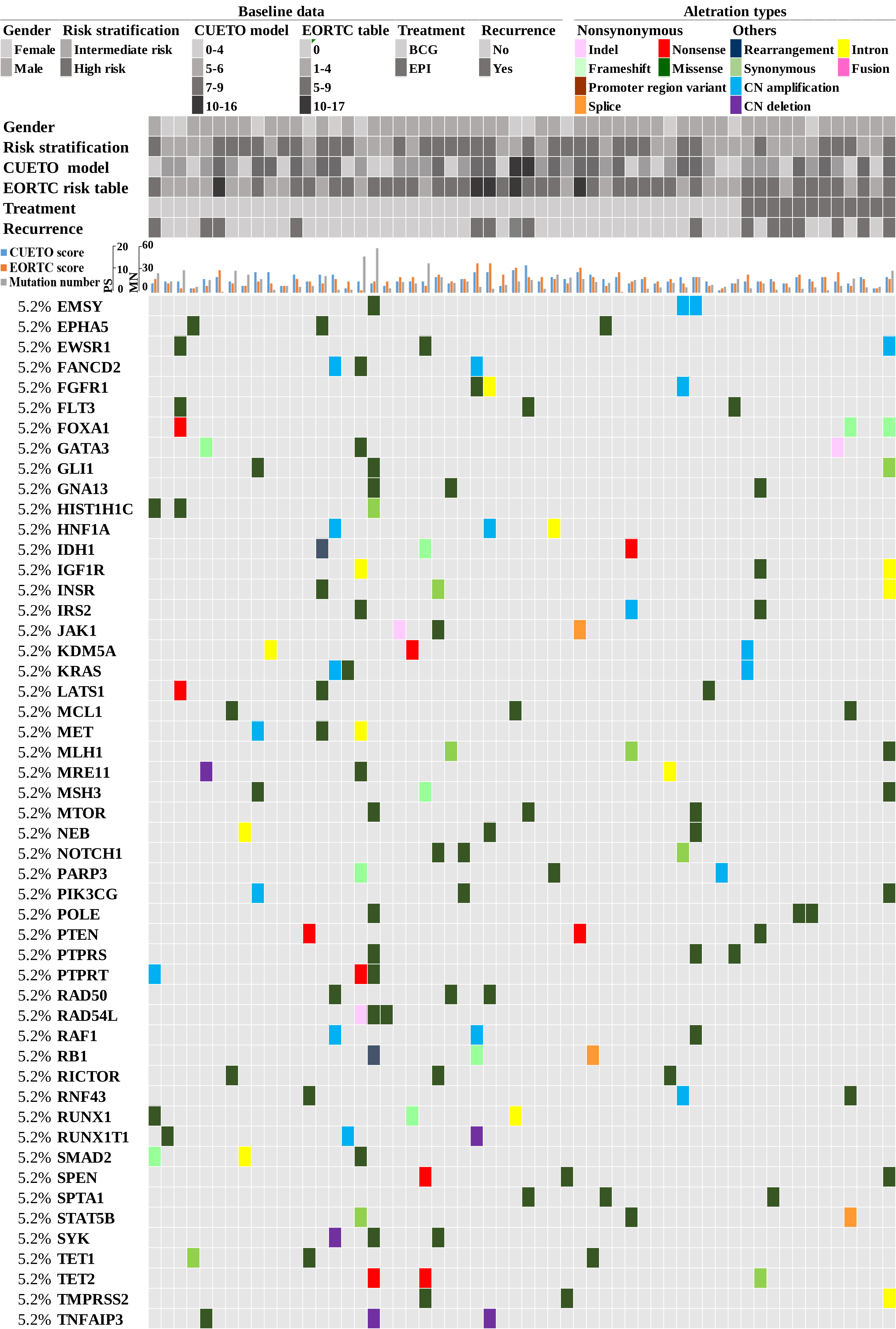

Baseline data
Aletration types
Gender
Female
Male
Risk stratification
Intermediate risk
High risk
CUETO model
0-4
5-6
7-9
10-16
EORTC table
0
1-4
5-9
10-17
Treatment
BCG
EPI
Recurrence
No
Yes
Nonsynonymous
Indel
Nonsense
Frameshift
Missense
Promoter region variant
Splice
Others
Rearrangement
Intron
Synonymous
Fusion
CN amplification
CN deletion
Gender
Risk stratification
CUETO model
EORTC risk table
Treatment
Recurrence
CUETO score
EORTC score
Mutation number
PS
MN
20
10
0
60
30
0
5.2% EMSY
5.2% EPHA5
5.2% EWSR1
5.2% FANCD2
5.2% FGFR1
5.2% FLT3
5.2% FOXA1
5.2% GATA3
5.2% GLI1
5.2% GNA13
5.2% HIST1H1C
5.2% HNF1A
5.2% IDH1
5.2% IGF1R
5.2% INSR
5.2% IRS2
5.2% JAK1
5.2% KDM5A
5.2% KRAS
5.2% LATS1
5.2% MCL1
5.2% MET
5.2% MLH1
5.2% MRE11
5.2% MSH3
5.2% MTOR
5.2% NEB
5.2% NOTCH1
5.2% PARP3
5.2% PIK3CG
5.2% POLE
5.2% PTEN
5.2% PTPRS
5.2% PTPRT
5.2% RAD50
5.2% RAD54L
5.2% RAF1
5.2% RB1
5.2% RICTOR
5.2% RNF43
5.2% RUNX1
5.2% RUNX1T1
5.2% SMAD2
5.2% SPEN
5.2% SPTA1
5.2% STAT5B
5.2% SYK
5.2% TET1
5.2% TET2
5.2% TMPRSS2
5.2% TNFAIP3

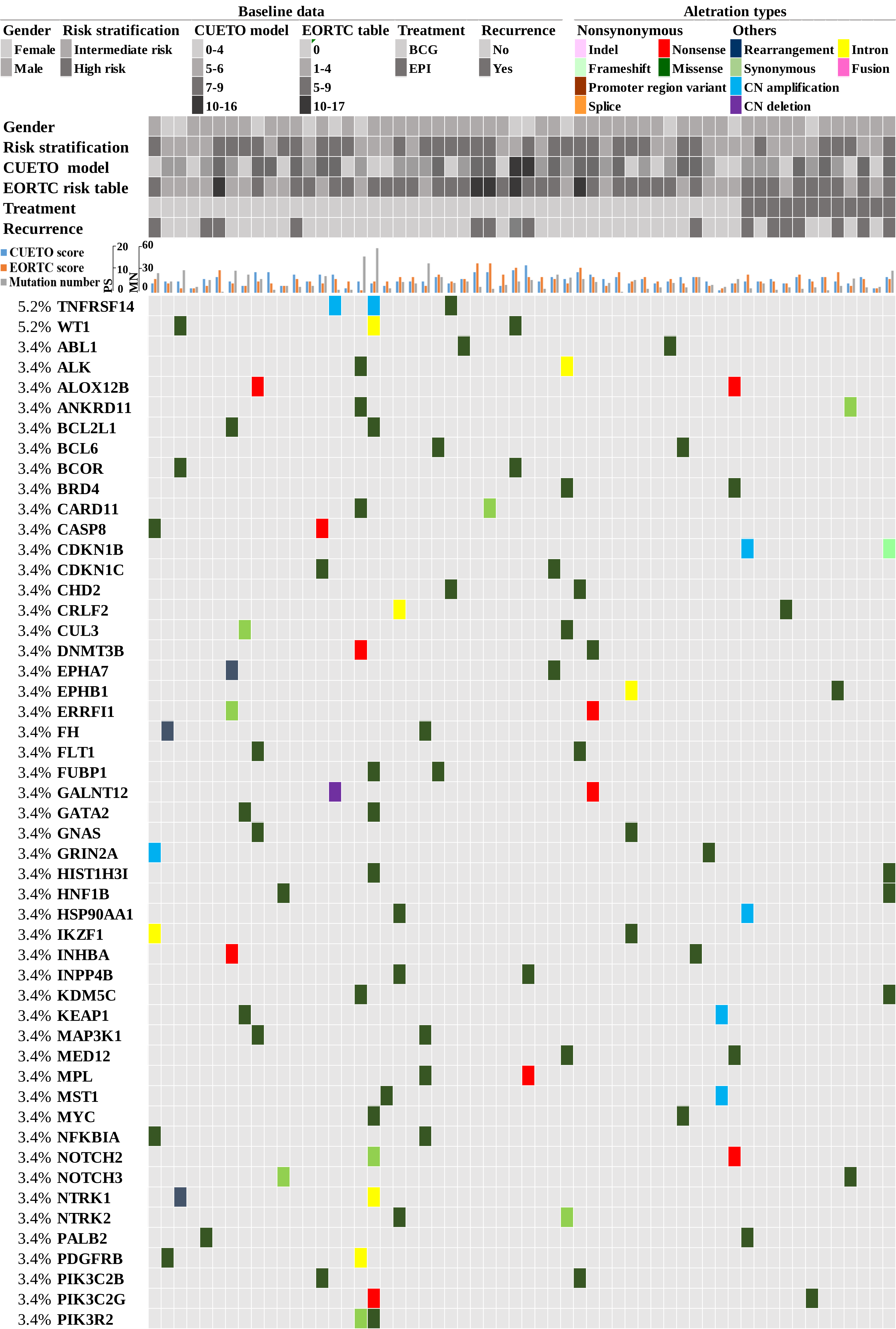
Baseline data
Aletration types
Gender
Female
Male
Risk stratification
Intermediate risk
High risk
CUETO model
0-4
5-6
7-9
10-16
EORTC table
0
1-4
5-9
10-17
Treatment
BCG
EPI
Recurrence
No
Yes
Nonsynonymous
Indel
Frameshift
Nonsense
Missense
Promoter region variant
Splice
Others
Rearrangement
Synonymous
CN amplification
CN deletion
Intron
Fusion
Gender
Risk stratification
CUETO model
EORTC risk table
Treatment
Recurrence
CUETO score
EORTC score
Mutation number
PS
MN
20
10
0
60
30
0
5.2% TNFRSF14
5.2% WT1
3.4% ABL1
3.4% ALK
3.4% ALOX12B
3.4% ANKRD11
3.4% BCL2L1
3.4% BCL6
3.4% BCOR
3.4% BRD4
3.4% CARD11
3.4% CASP8
3.4% CDKN1B
3.4% CDKN1C
3.4% CHD2
3.4% CRLF2
3.4% CUL3
3.4% DNMT3B
3.4% EPHA7
3.4% EPHB1
3.4% ERRFI1
3.4% FH
3.4% FLT1
3.4% FUBP1
3.4% GALNT12
3.4% GATA2
3.4% GNAS
3.4% GRIN2A
3.4% HIST1H3I
3.4% HNF1B
3.4% HSP90AA1
3.4% IKZF1
3.4% INHBA
3.4% INPP4B
3.4% KDM5C
3.4% KEAP1
3.4% MAP3K1
3.4% MED12
3.4% MPL
3.4% MST1
3.4% MYC
3.4% NFKBIA
3.4% NOTCH2
3.4% NOTCH3
3.4% NTRK1
3.4% NTRK2
3.4% PALB2
3.4% PDGFRB
3.4% PIK3C2B
3.4% PIK3C2G
3.4% PIK3R2

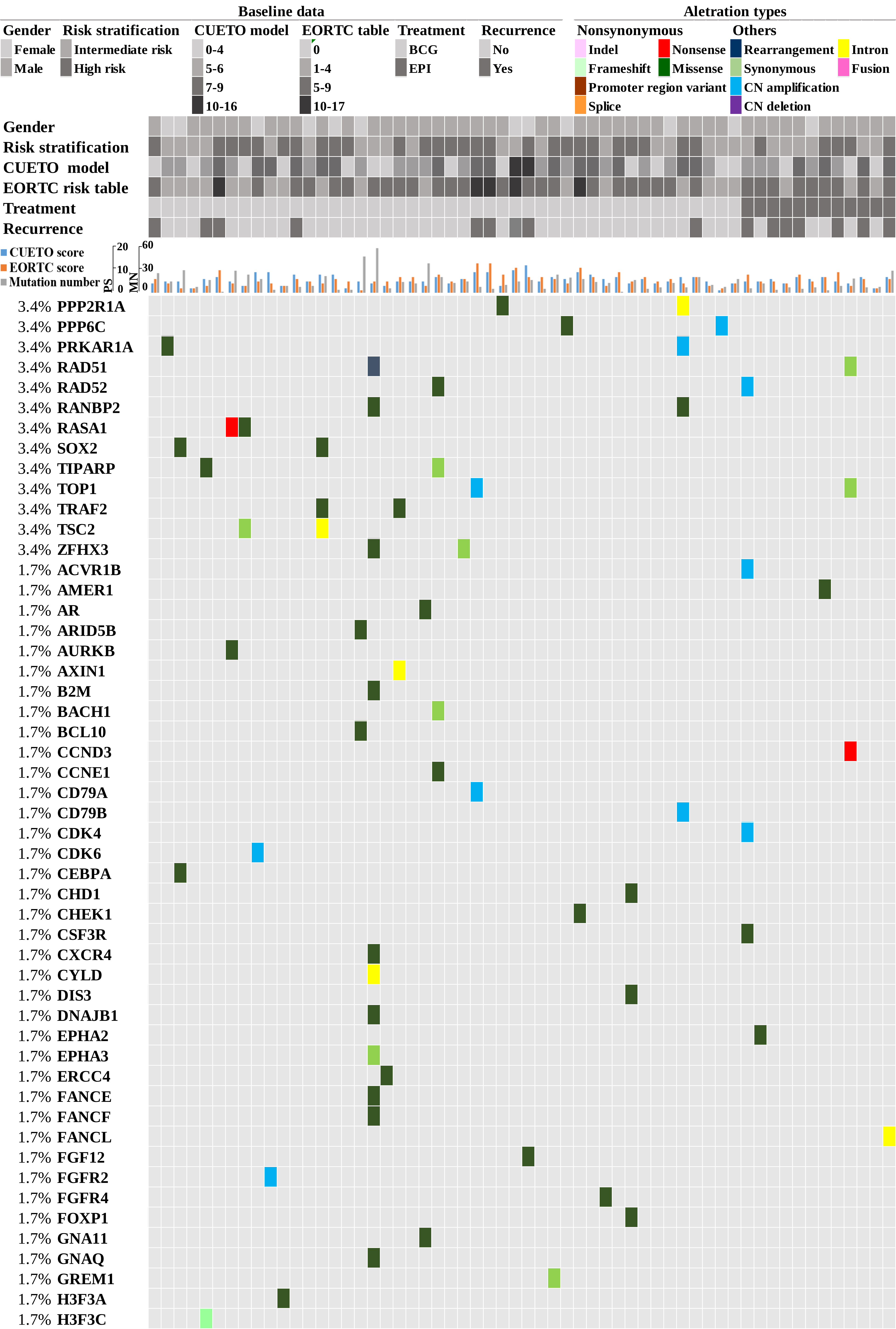
Baseline data
Aletration types
Gender
Female
Male
Risk stratification
Intermediate risk
High risk
CUETO model
0-4
5-6
7-9
10-16
EORTC table
0
1-4
5-9
10-17
Treatment
BCG
EPI
Recurrence
No
Yes
Nonsynonymous
Indel
Frameshift
Nonsense
Missense
Promoter region variant
Splice
Others
Rearrangement
Synonymous
CN amplification
CN deletion
Intron
Fusion
Gender
Risk stratification
CUETO model
EORTC risk table
Treatment
Recurrence
CUETO score
EORTC score
Mutation number
PS
MN
20
10
0
60
30
0
3.4% PPP2R1A
3.4% PPP6C
3.4% PRKAR1A
3.4% RAD51
3.4% RAD52
3.4% RANBP2
3.4% RASA1
3.4% SOX2
3.4% TIPARP
3.4% TOP1
3.4% TRAF2
3.4% TSC2
3.4% ZFHX3
1.7% ACVR1B
1.7% AMER1
1.7% AR
1.7% ARID5B
1.7% AURKB
1.7% AXIN1
1.7% B2M
1.7% BACH1
1.7% BCL10
1.7% CCND3
1.7% CCNE1
1.7% CD79A
1.7% CD79B
1.7% CDK4
1.7% CDK6
1.7% CEBPA
1.7% CHD1
1.7% CHEK1
1.7% CSF3R
1.7% CXCR4
1.7% CYLD
1.7% DIS3
1.7% DNAJB1
1.7% EPHA2
1.7% EPHA3
1.7% ERCC4
1.7% FANCE
1.7% FANCF
1.7% FANCL
1.7% FGF12
1.7% FGFR2
1.7% FGFR4
1.7% FOXP1
1.7% GNA11
1.7% GNAQ
1.7% GREM1
1.7% H3F3A
1.7% H3F3C

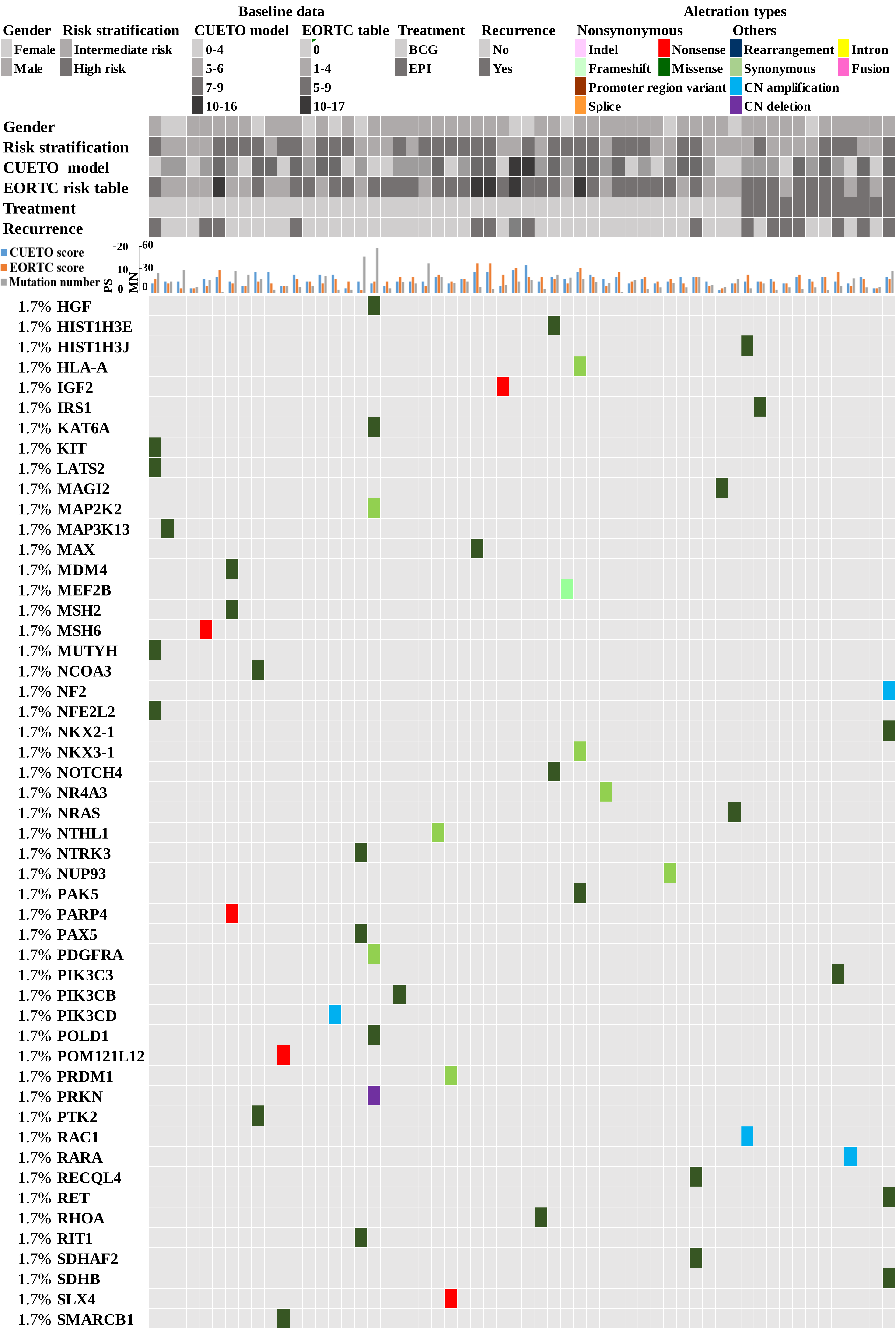
Baseline data
Aletration types
Gender
Female
Male
Risk stratification
Intermediate risk
High risk
CUETO model
0-4
5-6
7-9
10-16
EORTC table
0
1-4
5-9
10-17
Treatment
BCG
EPI
Recurrence
No
Yes
Nonsynonymous
Indel
Nonsense
Frameshift
Missense
Promoter region variant
Splice
Others
Rearrangement
Intron
Synonymous
Fusion
CN amplification
CN deletion
Gender
Risk stratification
CUETO model
EORTC risk table
Treatment
Recurrence
CUETO score
EORTC score
Mutation number
PS
MN
20
10
0
60
30
0
1.7% HGF
1.7% HIST1H3E
1.7% HIST1H3J
1.7% HLA-A
1.7% IGF2
1.7% IRS1
1.7% KAT6A
1.7% KIT
1.7% LATS2
1.7% MAGI2
1.7% MAP2K2
1.7% MAP3K13
1.7% MAX
1.7% MDM4
1.7% MEF2B
1.7% MSH2
1.7% MSH6
1.7% MUTYH
1.7% NCOA3
1.7% NF2
1.7% NFE2L2
1.7% NKX2-1
1.7% NKX3-1
1.7% NOTCH4
1.7% NR4A3
1.7% NRAS
1.7% NTHL1
1.7% NTRK3
1.7% NUP93
1.7% PAK5
1.7% PARP4
1.7% PAX5
1.7% PDGFRA
1.7% PIK3C3
1.7% PIK3CB
1.7% PIK3CD
1.7% POLD1
1.7% POM121L12
1.7% PRDM1
1.7% PRKN
1.7% PTK2
1.7% RAC1
1.7% RARA
1.7% RECQL4
1.7% RET
1.7% RHOA
1.7% RIT1
1.7% SDHAF2
1.7% SDHB
1.7% SLX4
1.7% SMARCB1

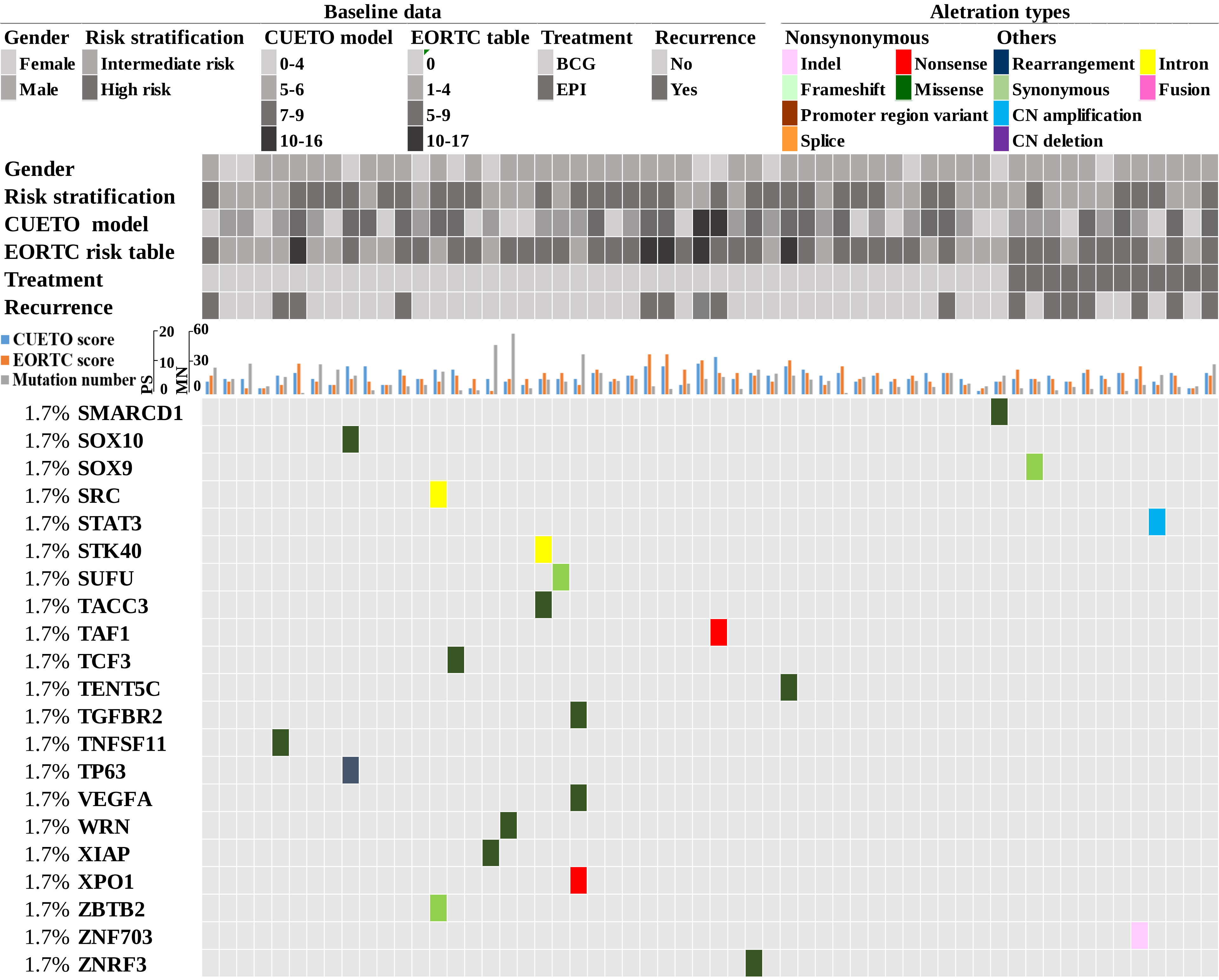
Baseline data
Aletration types
Gender
Female
Male
Risk stratification
Intermediate risk
High risk
CUETO model
0-4
5-6
7-9
10-16
EORTC table
0
1-4
5-9
10-17
Treatment
BCG
EPI
Recurrence
No
Yes
Nonsynonymous
Indel
Nonsense
Frameshift
Missense
Promoter region variant
Splice
Others
Rearrangement
Intron
Synonymous
Fusion
CN amplification
CN deletion
Gender
Risk stratification
CUETO model
EORTC risk table
Treatment
Recurrence
CUETO score
EORTC score
Mutation number
PS
MN
20
10
0
60
30
0
1.7% SMARCD1
1.7% SOX10
1.7% SOX9
1.7% SRC
1.7% STAT3
1.7% STK40
1.7% SUFU
1.7% TACC3
1.7% TAF1
1.7% TCF3
1.7% TENT5C
1.7% TGFBR2
1.7% TNFSF11
1.7% TP63
1.7% VEGFA
1.7% WRN
1.7% XIAP
1.7% XPO1
1.7% ZBTB2
1.7% ZNF703
1.7% ZNRF3
