# Supplementary figures and images for: Prognostic factors of non-muscle invasive bladder cancer: a study based on next-generation sequencing
Source: Cancer Cell Int. 2021 Jan 6;21:23. doi: 10.1186/s12935-020-01731-9 (PMC7789352; doi:10.1186/s12935-020-01731-9)

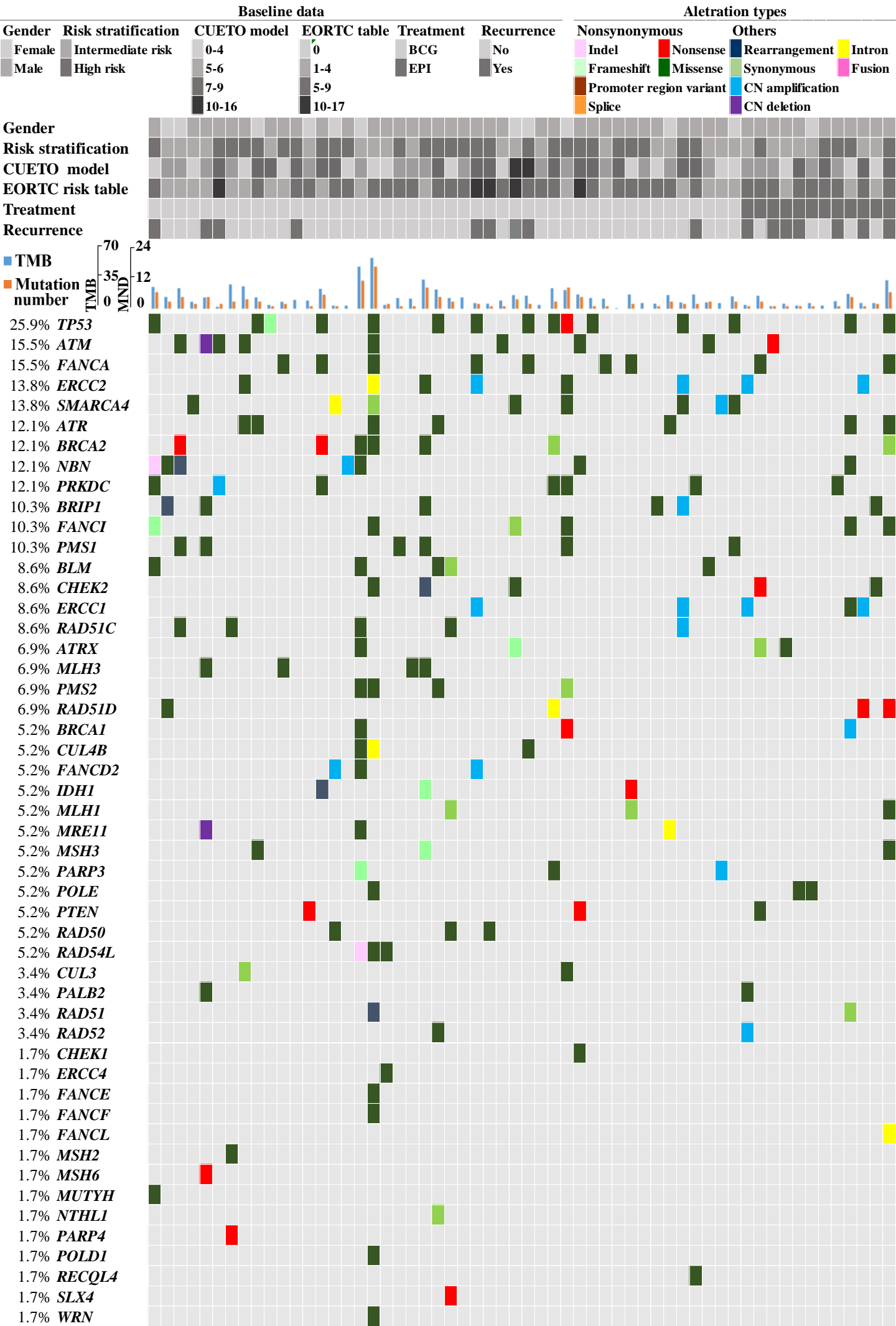

Supplement: Supplementary file 3 — Additional file 3: Figure S8. [file 12935_2020_1731_MOESM3_ESM.pdf]
